# Intrinsic Defects and Their Role in the Phase Transition of Na-Ion Anode Na_2_Ti_3_O_7_

**DOI:** 10.1021/acsaem.2c03466

**Published:** 2022-12-16

**Authors:** Yong-Seok Choi, Sara I. R. Costa, Nuria Tapia-Ruiz, David O. Scanlon

**Affiliations:** † Department of Materials Science and Engineering, Dankook University, Cheonan31116, South Korea; ‡ Department of Chemistry, University College London, 20 Gordon Street, LondonWC1H 0AJ, U.K.; § The Faraday Institution, Harwell Campus, DidcotOX11 0RA, U.K.; ∥ Thomas Young Centre, University College London, Gower Street, LondonWC1E 6BT, U.K.; ⊥ Department of Chemistry, Lancaster University, LancasterLA1 4YB, U.K.

**Keywords:** titanate anodes, sodium-ion battery, intrinsic defect chemistry, Schottky pair, electrical conductivity, phase transition, density functional theory

## Abstract

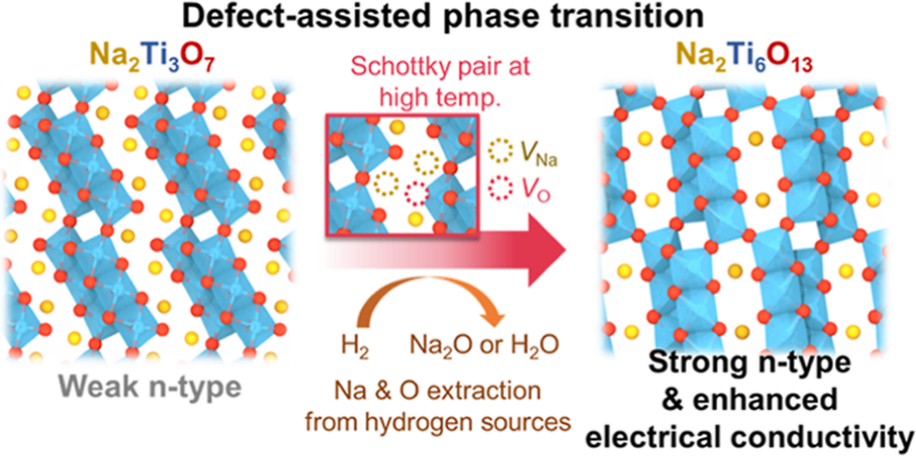

The development of high-power anode materials for Na-ion batteries is one of the primary obstacles due to the growing demands for their use in the smart grid. Despite the appealingly low cost and non-toxicity, Na_2_Ti_3_O_7_ suffers from low electrical conductivity and poor structural stability, which restricts its use in high-power applications. Viable approaches for overcoming these drawbacks reported to date are aliovalent doping and hydrogenation/hydrothermal treatments, both of which are closely intertwined with native defects. There is still a lack of knowledge, however, of the intrinsic defect chemistry of Na_2_Ti_3_O_7_, which impairs the rational design of high-power titanate anodes. Here, we report hybrid density functional theory calculations of the native defect chemistry of Na_2_Ti_3_O_7_. The defect calculations show that the insulating properties of Na_2_Ti_3_O_7_ arise from the Na and O Schottky disorder that act as major charge compensators. Under high-temperature hydrogenation treatment, these Schottky pairs of Na and O vacancies become dominant defects in Na_2_Ti_3_O_7_, triggering the spontaneous partial phase transition to Na_2_Ti_6_O_13_ and improving the electrical conductivity of the composite anode. Our findings provide an explanation on the interplay between intrinsic defects, structural phase transitions, and electrical conductivity, which can aid understanding of the properties of composite materials obtained from phase transitions.

## 1 Introduction

Owing to the ever-growing demands for sustainable energy storage devices in electric vehicles and grid applications, batteries composed of non-toxic and naturally abundant elements have increasingly attracted attention.^1,2^ Na-ion batteries (NIBs), taking advantage of Na being the fourth most earth-abundant element, have emerged as promising candidates for such applications. Together with cost-effective electrodes, the use of NIBs can bring a radical decrease in cost compared to the widely used Li-ion batteries, while ensuring sustainability. Sodium titanates (Na_2_Ti_*n*_O_2*n*+1_) are some of the most attractive anodes for sustainable NIBs because of the large abundance of its raw materials and non-toxicity.^3,4^ Among the sodium titanate family, Na_2_Ti_3_O_7_ has been reported to be suitable for NIBs due to its moderate theoretical capacity (177 mA h g^–1^)^3^ and low average working potential (0.3 vs Na^+^/Na).^5^ However, previous tests have shown that Na_2_Ti_3_O_7_ suffers from (i) low electrical conductivity and (ii) structural instability, which results in poor electrochemical performance, particularly at high charge/discharge rates. To enable the practical application of Na_2_Ti_3_O_7_, it is thus essential to develop proper strategies to overcome its drawbacks.

The low electrical conductivity of Na_2_Ti_3_O_7_ has been addressed by either introducing conductive matrices^6−10^ or aliovalent doping.^11−13^ Of these methods, the doping strategy has been widely utilized for Na_2_Ti_3_O_7_ with various dopants, including Nb^5+^,^11^ F^1–^,^13^ and lanthanides.^12^ Despite the extensive efforts on the doped Na_2_Ti_3_O_7_ anodes, the physics underlying the improved electrical conductivity are still under debate. For instance, previous X-ray photoelectron spectroscopy (XPS) and thermogravimetric analysis have suggested that n-type dopants improve the electrical conductivity by donating excess electrons and partially reducing Ti^4+^ to Ti^3+^.^11,13^ P-type lanthanide doping, on the other hand, is reported to cause a controversial behavior:^12^ XPS analyses on the Yb-doped Na_2_Ti_3_O_7_ showed additional peaks corresponding to Ti 2p doublets of Ti^3+^, suggesting the partial reduction of Ti^4+^. The authors interpreted this behavior to arise from oxygen vacancies created upon Yb^3+^ doping, which is arguable, as the so-formed oxygen vacancies would preferentially charge compensate the electron holes created by Yb^3+^ dopants, rather than donating extra electrons to Ti^4+^. From this perspective, Ti reduction can be an experimental artifact due to the difficulties in controlling synthesis conditions with low concentrations of dopants (5‰ Yb). The above example indicates that the experimental investigation on the doping effects of Na_2_Ti_3_O_7_ is challenging and could even lead to a wrong conclusion without prior knowledge of its defect chemistry. Therefore, to elucidate the defect chemistry of Na_2_Ti_3_O_7_ and guide proper dopant choice which can improve electrical conductivity, a computational approach should be carried out first on the intrinsic defects and their role in charge compensation mechanisms.

Other studies on the structural instability of Na_2_Ti_3_O_7_ have revealed that its structural degradation can be effectively alleviated when being mixed with another titanate, Na_2_Ti_6_O_13_.^14,15^ This strategy stems from the fact that materials with opposite properties can be mixed to complement respective drawbacks: Na_2_Ti_6_O_13_ has a higher degree of interconnection between Ti–O octahedra compared to that of Na_2_Ti_3_O_7_ and thus is more resistive to structural changes while having smaller space for Na intercalation. This indicates that the mixture of Na_2_Ti_3_O_7_ and Na_2_Ti_6_O_13_ will display improved cyclability, at the expense of the theoretical capacity. Recently, facile methods to synthesize this mixed anode have been suggested. When synthesized with hydrogen gas, some of the Na_2_Ti_3_O_7_ spontaneously transforms into Na_2_Ti_6_O_13_, which provides Na_2_Ti_3_O_7_/Na_2_Ti_6_O_13_ hybrid anodes without additional synthesis routes.^14,16,17^ This procedure typically includes two reactions of O and Na removal according to the following equations
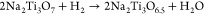
1
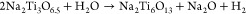
2

Above reactions in eqs 1 and 2 imply that O and Na vacancy defects formed during the synthesis condition are closely related to the spontaneous phase transition from Na_2_Ti_3_O_7_ to Na_2_Ti_6_O_13_. However, the interplay between defects and phase transition have been neglected to date, and the fundamental driving forces underlying the phase transition remain elusive.

To better understand the role of native defects on the above two issues of charge compensation mechanism and phase transition behavior, we perform computational analyses on the intrinsic defect chemistry in Na_2_Ti_3_O_7_. In particular, using the combined density functional theory (DFT) calculations with hybrid^18^ and PBEsol^19^ functionals, we replicate the high-temperature synthesis condition and investigate major defects formed during synthesis. The formation energies of the so-formed major defects are used to discuss the charge compensation behaviors and underlying conductivity mechanism, which can help in establishing a doping strategy for anodes with improved performance. Furthermore, the effect of intrinsic defects of Na_2_Ti_3_O_7_ on its atomic structure were studied to elucidate the origin of the spontaneous phase transition to Na_2_Ti_6_O_13_, which can potentially guide further optimization of anodes employing phase transitions.

## 2 Results and Discussion

### 2.1 Phase Stability

When investigating the intrinsic defect chemistry of a material, a key consideration is its thermodynamic stability under equilibrium growth conditions. In order for Na_2_Ti_3_O_7_ to be stable under certain environmental conditions, the chemical potentials of its elemental components, that is, μ_Na_, μ_Ti_, and μ_O_, have to satisfy certain conditions.^20^ First, to be in equilibrium with its elemental components, the free energy of formation (Δ*G*_f_) of Na_2_Ti_3_O_7_ should be expressed as the sum of the chemical potentials (μ) of each element according to the following equation
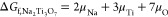
3

In addition, the chemical potentials should not allow the precipitation of other competing secondary phases under the growth condition, which forces chemical potentials to satisfy following relationships
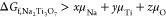
4

Equation 4 should be satisfied for all 17 stable competing phases of Na_*x*_Ti_*y*_O_*z*_ listed in Figure S1. Solving the simultaneous eqs 3 and 4 gives the chemical potential space of Na, Ti, and O where Na_2_Ti_3_O_7_ is thermodynamically stable.

**Figure 1:**
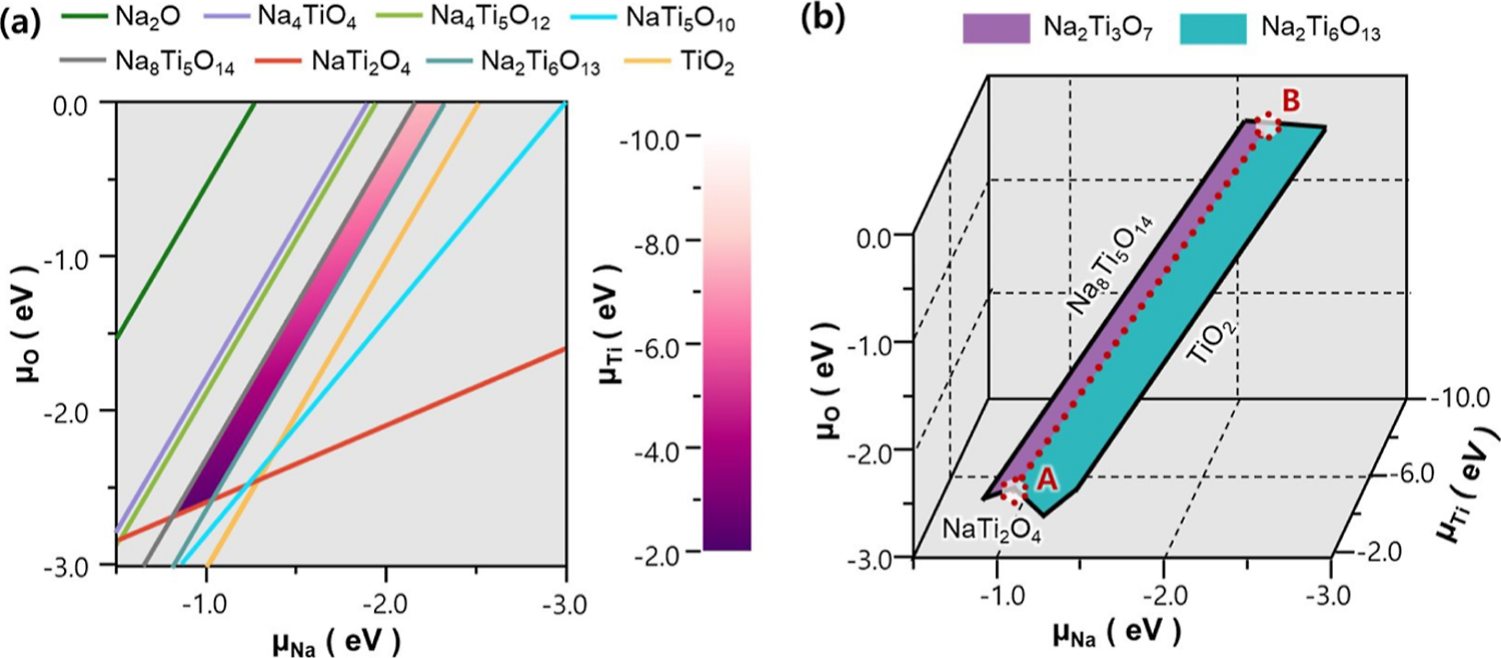
(a) Region of stability (purple area) for Na_2_Ti_3_O_7_ in the 2D space spanned by μ_Na_ and μ_O_, where the variation of μ_Ti_ is shown within the stability region. The (colored) lines indicate the limits imposed by competing phases. (b) Stability regions for Na_2_Ti_3_O_7_ (purple) and Na_2_Ti_6_O_13_ (cyan) in the 3D space spanned by μ_Na_, μ_Ti_, and μ_O_. Competing phases imposing the limits of stability regions are denoted at the boundaries. Chemical potential conditions where both Na_2_Ti_3_O_7_ and Na_2_Ti_6_O_13_ coexist are highlighted by the red dashed line.

The standard procedure^21^ to obtain such a stability region is to calculate formation energies at the athermal limit for all competing phases using the DFT total energy calculations. However, this method is unable to predict the stability fields of Na_2_Ti_3_O_7_ because this phase is stable only at elevated temperature^16^ and not at the athermal limit. To capture the stability region of Na_2_Ti_3_O_7_, it is thus necessary to take into account temperature effects on the phase stability. To this end, we evaluated the temperature-dependent Gibbs free energy of formation by incorporating the vibrational entropy under the quasi-harmonic approximation (see Figure S1 and Methods in Supporting Information). The calculated Gibbs free energy of formation was then used to plot the chemical potential region of stability for Na_2_Ti_3_O_7_ at a representative synthesis temperature of 1070 K (Figure 1a). The calculated stability region shows that Na_2_Ti_3_O_7_ is stable across a modest range of μ_Ti_, whereas stability regions for μ_Na_ and μ_O_ are restricted to relatively small ranges. This suggests that Na_2_Ti_3_O_7_ can tolerate a moderate amount of Ti deficiency, whereas small changes in Na or O stoichiometry, such as Na and O vacancies, can cause Na_2_Ti_3_O_7_ to be thermodynamically unstable and lead to the phase transition to other competing phases, for example Na_8_Ti_5_O_14_ and Na_2_Ti_6_O_13_.

The predicted chemical potential region can be further narrowed down depending on the synthesis conditions of interest. Among the synthesis methods available for Na_2_Ti_3_O_7_ anode, we selected hydrogenation treatment, which is reported to be effective in synthesizing high-performance Na_2_Ti_3_O_7_ anode.^14,16,17^ The key principle of this method is employing the partial phase transition from Na_2_Ti_3_O_7_ to Na_2_Ti_6_O_13_ during synthesis, which alleviates the structural instability inherent to Na_2_Ti_3_O_7_ and enhances the rate performance of the resultant mixture anode. To identify the chemical potential conditions corresponding to the hydrogenation treatment, we exploited the experimental observation that both Na_2_Ti_3_O_7_ and Na_2_Ti_6_O_13_ phases coexist after hydrogenation treatment: this phenomenon implies that the chemical potential condition during such a synthesis process allows both phases to be stable and thus should be in the space where two stability regions of Na_2_Ti_3_O_7_ and Na_2_Ti_6_O_13_ are overlapping. Based on this concept, we searched the chemical potential regions corresponding to hydrogenation treatment by plotting the stability fields of Na_2_Ti_6_O_13_ (Figure S3) and superimposed it with the region of stability for Na_2_Ti_3_O_7_ (Figure 1b). The result shows that both phases, Na_2_Ti_3_O_7_ and Na_2_Ti_6_O_13_, are thermodynamically stable when chemical potentials correspond to a dividing line between the two stability regions of Na_2_Ti_3_O_7_ and Na_2_Ti_6_O_13_ (see the red dashed line in Figure 1b). In this regard, we assume that these sets of chemical potentials represent a good approximation of the chemical environment of hydrogenation treatment. Note that the dividing line between Na_2_Ti_3_O_7_ and Na_2_Ti_6_O_13_ is characterized by an extremely narrow chemical potential region, which suggests that a small variation in Na, Ti, or O stoichiometries during such synthesis conditions can cause Na_2_Ti_3_O_7_ to become thermodynamically less stable than its most competitive secondary phase, Na_2_Ti_6_O_13_ and vice versa. Further analyses on the atomic rearrangements during such phase transition will give fundamental understanding on the origin of Na_2_Ti_6_O_13_ formed during hydrogenation treatment. In the following, we will discuss how the structural changes from Na_2_Ti_3_O_7_ to Na_2_Ti_6_O_13_ can be aided by intrinsic defects, which will be addressed under two representative conditions of oxygen-poor/metal-rich and oxygen-rich/metal-poor [(μ_Na_, μ_Ti_, μ_O_) = (−1.05, −2.44, −2.55) and (−2.32, −7.54, 0.0), respectively, as denoted by A and B in Figure 1b].

### 2.2 Intrinsic Defect Chemistry

**Figure 2:**
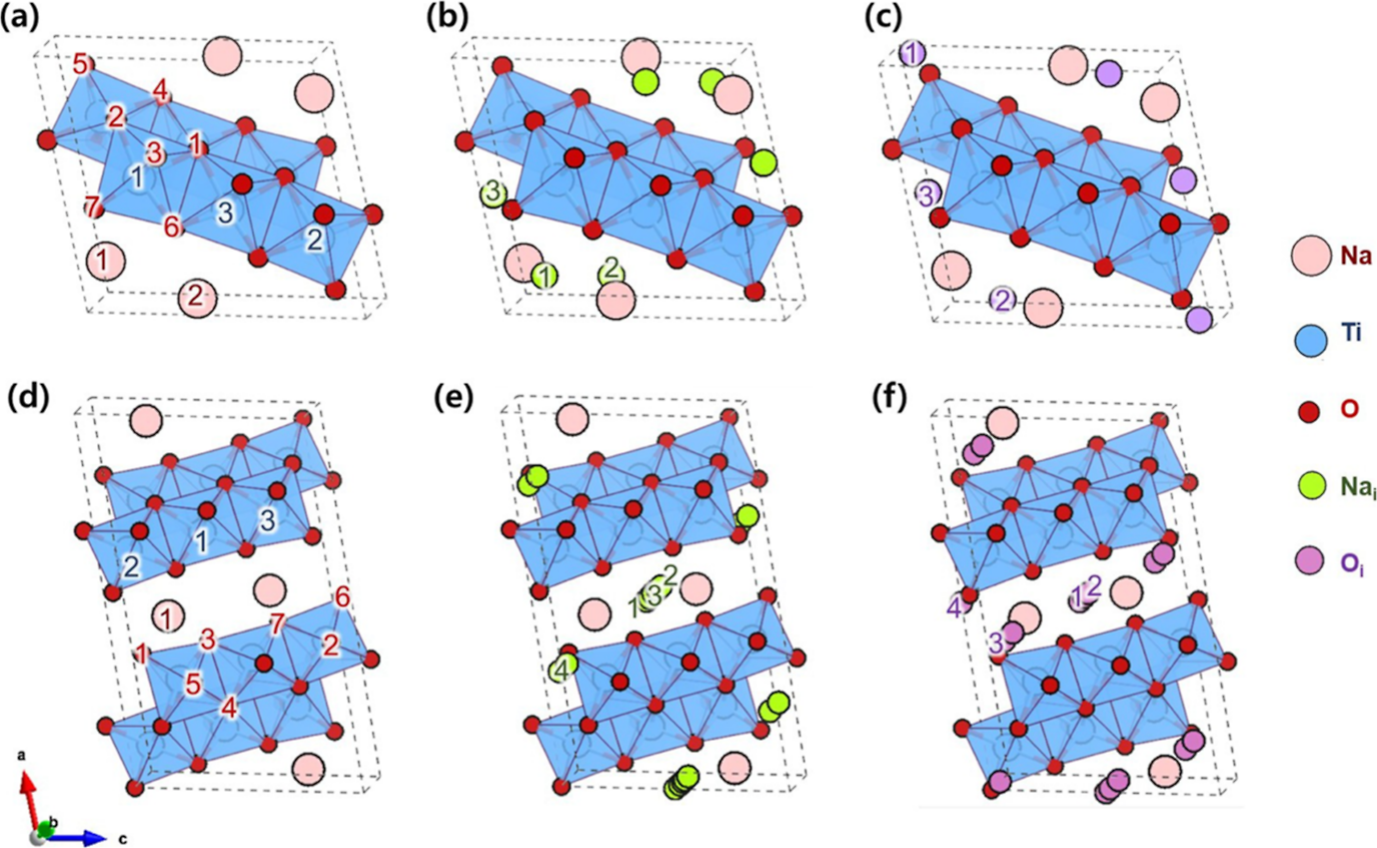
Crystal structures of conventional (a–c) Na_2_Ti_3_O_7_ (*P*2_1_/*m*) and (d–f) Na_2_Ti_6_O_13_ (*C*2/*m*), showing the symmetry unique defect sites used in defect calculations.

Close examination of the atomic structures of Na_2_Ti_3_O_7_ and Na_2_Ti_6_O_13_ reveals that, in addition to the similarity in thermodynamic stability noted above, both structures exhibit similar crystalline structure. Specifically, the two structures share the same framework of zigzag Ti–O layers, where TiO_6_ octahedra are stacked along the *b*-axis direction (Figure 2). Only slight differences are observed in the coordination environments of Na and O. Under such similar atomic configurations, small concentration of point defects can destabilize the long-range ordering of atomic frameworks, resulting in the phase transition from one to another. In order to gain insights into the role of intrinsic defects in phase transition behavior, we first created eight different point defects: *V*_Na_, *V*_Ti_, *V*_O_, Ti_Na_, Na_Ti_, Na_i_, O_i_, and Na_Ti_. For all selected vacancies and antisite defects, we have explicitly considered all symmetry inequivalent positions, whereas interstitial defects are modeled by adding the Na or O atom in interstitial sites predicted from the Voronoi polyhedron method (see Figure 2 and Table 1), as discussed in the Methods section.

**Table 1:** Fractional Coordinates of Symmetry Unique Na, Ti, and O Sites in Conventional Na_2_Ti_3_O_7_ and Na_2_Ti_6_O_13_ Structures Relaxed Using HSE06 Functionals^a^

	coordinates			
elements	*X*	*y*	*z*	Wyckoff position
Na_2_Ti_3_O_7_				
Na(1)	0.31876	0.25000	0.41807	2e
Na(2)	0.49705	0.75000	0.15963	2e
Ti(1)	0.96921	0.25000	0.72144	2e
Ti(2)	0.75087	0.25000	0.32208	2e
Ti(3)	0.85454	0.25000	0.01653	2e
O(1)	0.08436	1.25000	0.95565	2e
O(2)	0.19647	1.25000	0.68297	2e
O(3)	0.01605	0.75000	0.75463	2e
O(4)	0.32809	0.75000	0.90805	2e
O(5)	0.44869	0.75000	0.65397	2e
O(6)	0.22043	0.75000	0.18784	2e
O(7)	0.15194	0.75000	0.46355	2e
Na_i_(1)	0.59233	0.25207	0.60157	4f
Na_i_(2)	0.63901	0.75335	0.88681	4f
Na_i_(3)	0.94621	0.76342	0.52454	4f
O_i_(1)	0.48897	0.25014	0.56258	4f
O_i_(2)	0.57302	0.75171	0.74492	4f
O_i_(3)	0.05589	0.75001	0.48622	2e
Na_2_Ti_6_O_13_				
Na(1)	0.46264	1.00000	0.26783	4i
Ti(1)	0.66642	0.50000	0.43577	4i
Ti(2)	0.61524	0.50000	0.09783	4i
Ti(3)	0.72854	0.50000	0.76881	4i
O(1)	0.33651	0.50000	0.08484	4i
O(2)	0.35615	1.00000	0.88024	4i
O(3)	0.37436	0.50000	0.38412	4i
O(4)	0.20331	0.50000	0.42923	4i
O(5)	0.23954	1.00000	0.24808	2a
O(6)	0.50000	0.50000	1.00000	4i
O(7)	0.43163	0.50000	0.70740	4i
Na_i_(1)	0.00101	0.00007	0.49789	4i
Na_i_(2)	0.00014	0.43941	0.49935	8j
Na_i_(3)	0.00082	0.75027	0.50008	8j
Na_i_(4)	0.20393	0.76583	0.94977	8j
O_i_(1)	0.00000	0.00007	0.50005	2c
O_i_(2)	0.00025	0.75003	0.50005	4h
O_i_(3)	0.11051	0.74995	0.85874	8j
O_i_(4)	0.50000	0.003429	0.00000	4g

a Wyckoff position for each site is also included for reference.

**Figure 3:**
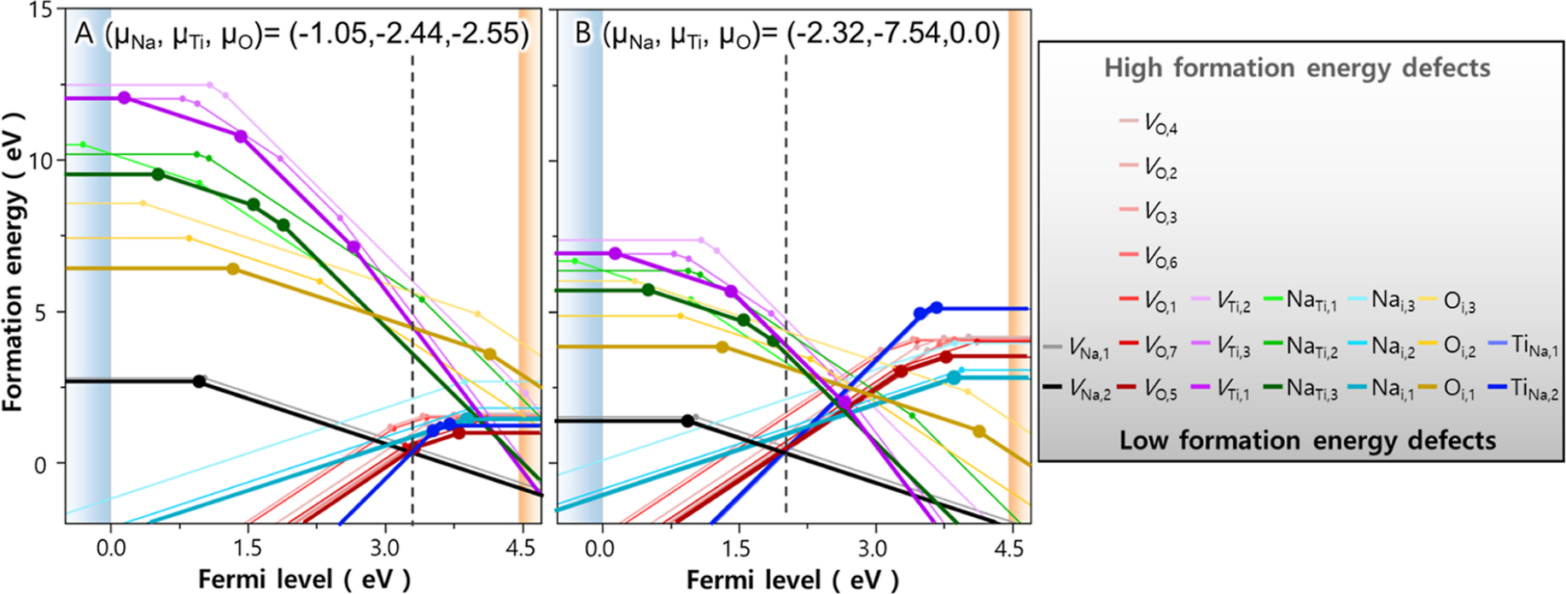
Transition level diagram of Na_2_Ti_3_O_7_ plotted under two different sets of elemental chemical potentials selected in Figure 1b, showing defect formation energies as a function of Fermi energy. Vertical dashed lines denote self-consistent (SC) Fermi energy at 1070 K where the concentration of excess electrons and holes satisfy charge neutrality.

In each of oxygen-poor and oxygen-rich conditions (A and B in Figure 1b), we plotted a defect transition level diagram of Na_2_Ti_3_O_7_ (Figure 3), which shows defect formation energies as a function of Fermi energy. Overall, most intrinsic defects are characterized by deep ε(0/−) or ε(+/0) transition levels and act as trap levels (Table 2). This is especially the case for the dominant defects (*V*_Na_, *V*_O_, Ti_Na_, and Na_i_) with a formation energy less than 3.0 eV: ε(0/−) transition levels for the acceptor defects of *V*_Na_ are above valence band maximum (VBM) by more than 0.97 eV, whereas ε(+/0) and ε(2+/0) transition levels for donor defects of *V*_O_, Ti_Na_, and Na_i_ are greater than 0.45 eV with respect to conduction band minimum (CBM). Such deep transition levels suggest that all acceptor and donor defects are too deep to be thermally activated at room temperature and thus act as carrier traps that reduce the carrier concentration and lifetime. Owing to such deep transition levels of intrinsic defects, defect hole and electron states tend to be localized on the nearest coordinating atoms (Table 3). For *V*_Na_, it represents hole polaron localized on neighboring oxygens, particularly those in site 5 (O_5_). In contrast, for the donor defects (*V*_O_, Ti_Na_, and Na_i_), all excess electrons are trapped over adjacent Ti^4+^ ions. Note that, unlike the other defects that have electron/hole polaron positioned neighboring atoms, *V*_Na_ shows preferential hole localization on O_5_. Such preferential hole localization plays an important role in defect pair formation and subsequent phase transition, which will be discussed in detail in Section 2.3.

**Table 2:** Thermodynamic Transition Levels (*c*) of Native Defects in Na_2_Ti_3_O_7_ Obtained with Respect to VBM and CBM

	*c* (vs VBM, eV)						*c* (vs CBM, eV)				
	(−3|−4)	(−2|−3)	(−1|−3)	(−1|−2)	(0|−2)	(0|−1)	(1|0)	(2|0)	(2|1)	(3|1)	(3|2)
*V*_Na,1_						1.02					
*V*_Na,2_						0.97					
*V*_O,1_							1.02		1.38		
*V*_O,2_							0.72		1.02		
*V*_O,3_							1.06		1.42		
*V*_O,4_							0.45		0.91		
*V*_O,5_							0.66		1.15		
*V*_O,6_							0.61		0.77		
*V*_O,7_							0.36		1.26		
*V*_Ti,1_	2.66		1.43			0.17					
*V*_Ti,2_	4.53	1.25			1.08						
*V*_Ti,3_	2.5	1.85		0.94		0.78					
Na_Ti,1_		2.3		0.97		–0.31					
Na_Ti,2_		3.4		1.07		0.94					
Na_Ti,3_		1.9		1.57		0.53					
Na_i,1_							0.59				
Na_i,2_							0.53				
Na_i,3_							0.61				
O_i,1_				4.13		1.33					
O_i,2_				2.28		0.85					
O_i,3_				4.01		0.35					
Ti_Na,1_								0.87			0.90
Ti_Na,2_							0.78			0.92	

**Table 3:** Localization of Polarons on Ions Adjacent to the Native Defects of Na_2_Ti_3_O_7_^a^

	polarons	constituents
Na_Ti,1_	0.864 (Ti3), 0.718 (O5), 0.446 (O6), 0.37 (O6)	superoxide (Ti–O–O)
Na_Ti,2_	0.703 (O2), 0.703 (O2), 0.737 (O5)	
Na_Ti,3_	0.647 (O5), 0.643 (O6), 0.302 (O7), 0.634 (O1), 0.274 (O4)	
O_i,1_	1.349 (interstitial), 0.111 (O5), 0.111 (O5), 0.13 (O5)	
O_i,2_	0.617 (interstitial), 0.792 (Ti2), 0.238 (O5)	superoxide (Ti–O–O)
O_i,3_	0.528 (interstitial), 0.843 (Ti1), 0.259 (O7)	superoxide (O–Ti–O)
*V*_Na,1_	0.706 (O5)	
*V*_Na,2_	0.733 (O5)	
*V*_Ti,1_	0.631 (O2), 0.66 (O6), 0.689 (O1), 0.534 (O7)	
*V*_Ti,2_	0.808 (O5), 0.726 (O2), 0.702 (O4), 0.727 (O2)	
*V*_Ti,3_	0.546 (O4), 0.665 (O1), 0.665 (O1), 0.692 (O6)	
Na_i,1_	0.902 (Ti1)	
Na_i,2_	0.891 (Ti1)	
Na_i,3_	0.879 (Ti1)	
Ti_Na,1_	0.987 (substitution), 0.912 (Ti1), 0.912 (Ti1)	
Ti_Na,2_	0.998 (substitution), 0.895 (Ti1), 0.893 (Ti2)	
*V*_O,1_	0.647 (Ti3), 0.647 (Ti3)	
*V*_O,2_	0.394 (Ti2), 0.658 (Ti1), 0.394 (Ti2)	
*V*_O,3_	0.671 (Ti1), 0.662 (Ti1)	
*V*_O,4_	0.274 (Ti2), 1.202 (Ti3)	
*V*_O,5_	0.849 (Ti1), 0.892 (Ti2)	
*V*_O,6_	0.625 (Ti1), 0.553 (Ti3)	
*V*_O,7_	1.014 (Ti1), 0.609 (Ti2)	

a The site of localized hole/electron polarons are denoted in parenthesis. Oxygen dimer species formed upon DFT structural optimizations are also shown for reference.

Further examination of acceptor defects shows that some defects, such as Na_Ti,1_, O_i,2_, and O_i,3_, lead to the formation of oxygen dimers (Table 3); in the case of Na_Ti,1_, the Na antisite reduces the coordination numbers of Ti–O, while creating hole polarons on neighboring oxygens. This destabilizes an oxygen atom coordinated by a single Ti atom (O_5_) and detaches it from the Ti–O framework to form an oxygen dimer with Ti–O–O coordination. O_i,2_ and O_i,3_, on the other hand, create oxygen dimers by bonding with neighboring low-coordinated oxygens of O_5_ and O_7_. These oxygen dimers are referred to as peroxide (O^2*–*^), superoxide (O^*–*^), or molecular oxygen (O_2_), depending on the number of localized holes. Although it is not straightforward to distinguish the oxygen redox species, such procedure can be done based on the length of O–O bonds (*d*_OO_) and the sum of magnetic moments (μ_i_) of constituent oxygens μ_O_: 1.4 Å ≤ *d*_OO_ ≤ 1.5 Å and Σ*|*μ_i_*|* ≤ 0.3 for peroxides and 1.25 Å ≤ *d*_OO_ ≤ 1.4 Å and 0.3 ≤ Σ*|*μ_i_*|* ≤ 1.4 for superoxides.^22,23^ Based on these criteria, all oxygen dimers formed upon the presence of Na_Ti,1_, O_i,2_, and O_i,3_ are identified to be superoxides. According to the polaron localization as presented in Table 3, the formation of superoxides is expected to proceed through the electron redistribution from O^2–^–Ti^4+^ to O^–^–Ti^3+^, which is then followed by the formation of O–O bonds with oxidation states of −0.5 (i.e., O^0.5–^–O^0.5–^–Ti^3+^ or O^0.5–^–Ti^3+^–O^0.5–^). The above results indicate that oxygens coordinated by few Ti atoms are susceptible to form superoxide species under the presence of Na_Ti_ and O_i_ defects. This trend can also be confirmed from the fact that superoxide dimers are less likely to form in Na_2_Ti_6_O_13_ where oxygens are relatively highly coordinated than Na_2_Ti_3_O_7_ (Table S3). However, the amount of superoxide oxygens is expected to be low for both Na_2_Ti_3_O_7_ and Na_2_Ti_6_O_13_ because the formation energies of Na_Ti_ and O_i_ in these materials are relatively higher than other defects. Another finding from Figure 3 is that, under both oxygen-poor and oxygen-rich conditions, Na vacancy in site 2 (*V*_Na,2_) is the most favorable native acceptor defect for Fermi energies within the band gap, except a small range close to CBM under oxygen-rich conditions. It is an acceptor-like defect throughout the majority of band gap and thus would compensate any donor defects created upon synthesis. In the case of donor defect of Na_2_Ti_3_O_7_, on the other hand, dominant defect species changes depending on chemical potential conditions. To better understand the charge compensation mechanisms of Na_2_Ti_3_O_7_, therefore, we compared the transition level diagrams calculated under A and B chemical potential sets as shown in Figure 1b, which correspond to relatively oxygen-poor and oxygen-rich conditions during hydrogenation treatment, respectively.

When examining the transition level diagram under oxygen-poor conditions, negatively charged Na vacancies (*V*_Na_^′^ in terms of Kröger–Vink notation) will most likely to be compensated by donor defects with the lowest formation energy, that is, antisite defect of Ti_Na_^•••^ in site 2 when the Fermi energy is near the VBM, and by oxygen vacancies in site 5 (*V*_O_^•^) if the Fermi energy is close to the CBM. Under thermodynamic equilibrium, these charge compensations pin the Fermi energy in the middle of band gap [referred to as self-consistent (SC) Fermi energy] to satisfy the condition of charge neutrality. The calculated SC Fermi energy for oxygen-poor conditions is pinned at 3.36 eV, which causes NTO to be a weak n-type material. Close examination on the transition level diagram shows that, at the SC Fermi level, three types of defects, that is, *V*_Na_^′^, *V*_O_^•^, and Ti_Na_^•••^, are expected to be dominant ones with low formation energies of 0.29, 0.51, and 0.57 eV, respectively.

Under oxygen-rich conditions, on the other hand, the overall formation energies of donor defects are increased, whereas those of acceptor defects are lowered. Such changes in formation energies increase the concentration of acceptor defects and associated hole polarons in Na_2_Ti_3_O_7_, causing the SC Fermi level to be shifted toward VBM and fixed at 2.07 eV. Despite these changes in defect formation energies, the most favorable defects at the SC Fermi level are similar to those under oxygen-poor conditions, that is, *V*_Na_^′^, *V*_O_^••^, and Ti_Na_^•••^, with formation energies at the SC Fermi level corresponding to 0.31, 0.52, and 0.52 eV, respectively. The above defect analyses on the two different chemical potential conditions suggest that Na_2_Ti_3_O_7_ synthesized under hydrogenation treatment is a weak n-type material, with its SC Fermi level being positioned from the middle of band gap (Figure 3b) to slightly close to CBM (Figure 3a). This suggests that n-type doping is predicted to be more effective in increasing the charge carrier density and thus improving electrical conductivity of Na_2_Ti_3_O_7_. P-type doping, on the other hand, is expected to cause the formation of additional donor defects that charge compensate holes created by dopants. The potential main “killer” defects are O vacancies (*V*_O_^•^ and *V*_O_^••^) and antisite defect of Ti_Na_^•••^ with low formation energies, which can result in lattice distortions of anodes as observed from previous experiments.^12^

Another important finding from Figure 3 is that, regardless of the chemical potential conditions, dominant intrinsic defects are commonly found to be *V*_Na_^′^, *V*_O_^•^, *V*_O_^••^, and Ti_Na_^•••^. This suggests two different types of charge-compensating mechanisms to occur during hydrogenation treatment: (1) Schottky pairs of Na and O vacancies (2*V*_Na_^′^ + *V*_O_^••^ or *V*_Na_^′^ + *V*_O_^•^) and (2) Ti antisite and Na vacancy pair (Ti_Na_^•••^ + 3*V*_Na_^′^). Depending on which defect pairs are dominant in Na_2_Ti_3_O_7_, it can undergo differing atomic rearrangements and associated phase transition. To verify the relationship between the intrinsic defects and phase transition behavior, it is thus necessary to quantitatively evaluate the defect concentrations formed in Na_2_Ti_3_O_7_. For this purpose, defect concentrations at the SC Fermi energy as shown in Figure 3 are calculated at a representative synthesis temperature of 1070 K. In this study, we assumed that the defects formed in Na_2_Ti_3_O_7_ during heat treatment are “frozen in” upon quenching and thus the defect concentrations calculated at high temperature can represent ones at room temperature. This argument can be justified based on the fact that, even if certain high-energy defects formed at high temperature are unstable after cooling down the system, kinetic barriers for their rearrangements are large enough to preserve them at room temperature.^24^

**Figure 4:**
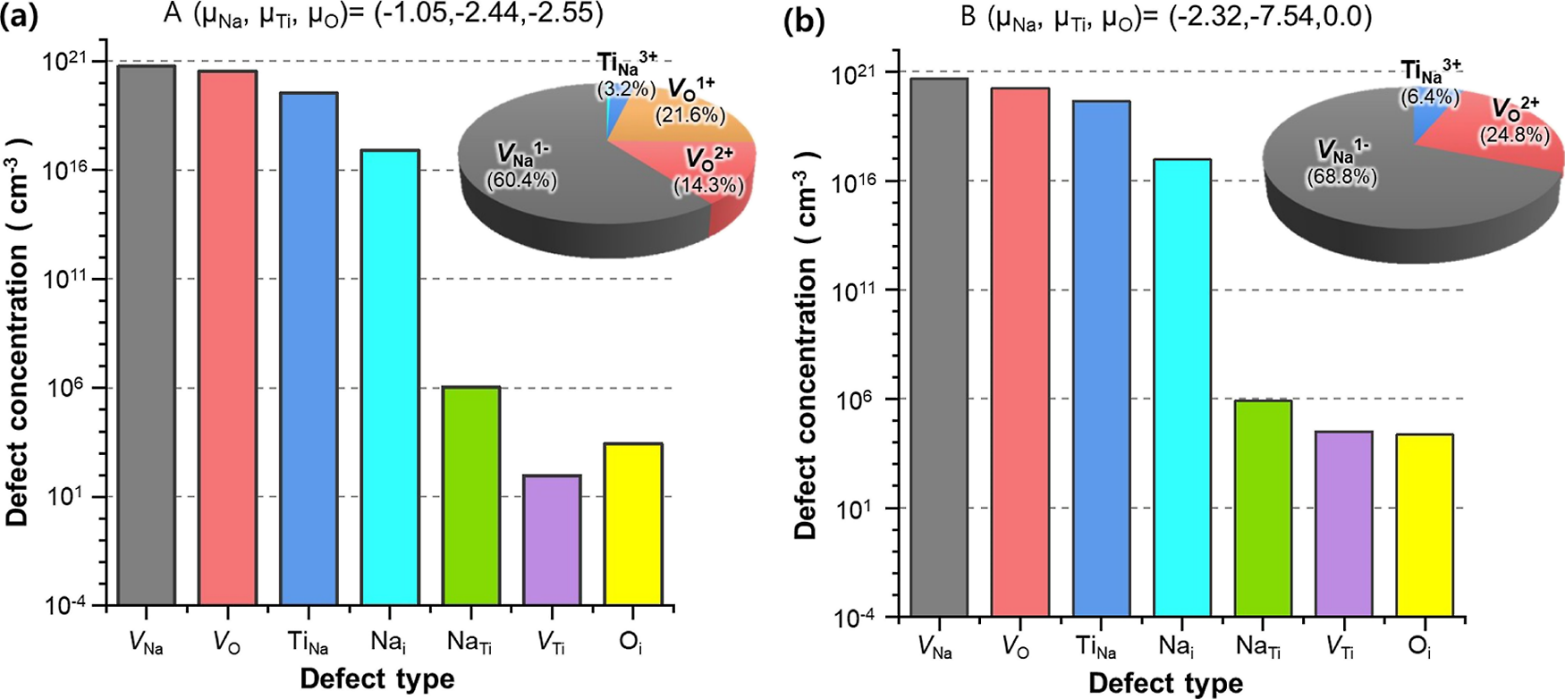
Equilibrium defect concentrations of Na_2_Ti_3_O_7_ at the elemental chemical potential conditions of (a) A and (b) B in Figure 1b. Bar graphs are plotted in the logarithmic scale, whereas pie charts in the insets are in the linear scale. All defect concentrations are calculated at 1070 K.

Figure 4 shows the equilibrium concentrations of various defects predicted at two different sets of chemical potentials of A and B in Figure 1. Overall, defects with lower formation energy at SC Fermi level as shown in Figure 3 exhibit higher defect concentrations. Of all defects considered, those other than *V*_Na_, *V*_O_, and Ti_Na_ are negligible because their concentrations are lower than *V*_Na_ by more than 3 orders of magnitude. Close examination on the concentrations of three major defects (*V*_Na_, *V*_O_, and Ti_Na_) shows that *V*_Na_ and *V*_O_ comprise a major portion (94–97%) of intrinsic defects (see the insets of Figure 4), where the ratio of *V*_Na_ and *V*_O_ is around 2:1. Compared to *V*_Na_ and *V*_O_, the amount of Ti_Na_ (3–6%) is relatively minor. These defect concentrations suggest that, among all native defects, Schottky pairs of Na and O vacancies (2*V*_Na_^′^ + *V*_O_^••^ or *V*_Na_^′^ + *V*_O_^•^) act as a main charge-compensating defects and thus are primarily responsible for the low electrical conductivity of Na_2_Ti_3_O_7_.

By converting the predicted defect concentrations of Schottky pairs into Na (*x*_Na_) and O (*x*_O_) stoichiometries of Na_2_Ti_3_O_7_, we can further analyze potential influence of intrinsic defects on phase transition behaviors. For a given Na_2_Ti_3_O_7_ with a volume per unit chemical formula of 145.41 Å^3^ (Table S1), Na and O stoichiometries under oxygen-poor and oxygen-rich conditions are *x*_Na,n_ = 1.92, *x*_Na,p_ = 1.93, *x*_O,n_ = 6.95, and *x*_O,p_ = 6.97. It is noted that, in both chemical potential conditions, the deviation in stoichiometries of Na_2_Ti_3_O_7_ occurs toward secondary Na_2_Ti_6_O_13_. For instance, Na_1.93_Ti_3_O_6.97_ under oxygen-rich growth conditions can be expressed as Na_1.93_Ti_3_O_6.97_ → 0.035Na_2_Ti_6_O_13_ + 0.93Na_2_Ti_3_O_7_, whereas Na_1.92_Ti_3_O_6.95_ under oxygen-poor conditions can be split into Na_1.92_Ti_3_O_6.95_ → 0.04Na_2_Ti_6_O_13_ + 0.92Na_2_Ti_3_O_7_. This indicates that about 7–8% of bulk Na_2_Ti_3_O_7_ have the composition similar to Na_2_Ti_6_O_13_, implying the precipitation of secondary Na_2_Ti_6_O_13_ during the synthesis of Na_2_Ti_3_O_7_. The amount of precipitated Na_2_Ti_6_O_13_, however, will be lower than those (7–8%) predicted from Na stoichiometry because Na_i,3_ in Na_2_Ti_3_O_7_ may act as a trapping site that facilitates the clustering of Na_i_ and *V*_Na_ pairs, lowering the mobile Na population (see additional notes in the Supporting Information).

### 2.3 Phase Transition from Na_2_Ti_3_O_7_ to Na_2_Ti_6_O_13_

**Figure 5:**
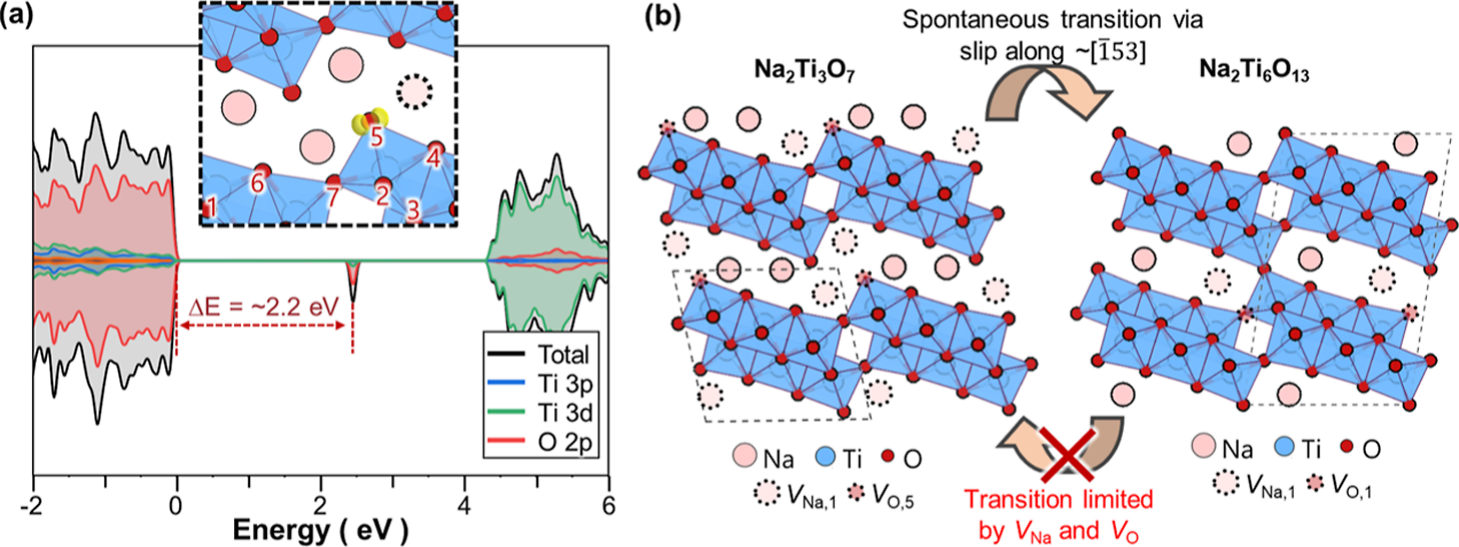
(a) Projected DOS calculated for Na_2_Ti_3_O_7_ after the removal of a Na atom from Na_2_. The inset shows the partial charge density for the hole band located 2.2 eV above the VBM. Type of oxygen sites are denoted as numbers in the inset. (b) Schematics of major intrinsic defects in Na_2_Ti_3_O_7_ Na_2_Ti_3_O_13_ and their role on the irreversible phase transition behavior.

To further understand the formation of Schottky pairs and how it affects the phase transition behavior, we modeled the Na vacancy defects and analyzed its relation to the O vacancy. Figure 5a shows the density of states (DOS) after removing a Na atom from site 2 as presented in Table 1. The DOS values reveal that, among O 2p electron population that predominantly occupies VBM, some electrons were extracted along with the Na atom, forming a hole defect state in the middle of the band gap. The hole defect state is strongly localized in O 2p electron, as revealed by the large offset of 2.2 eV from the top of VBM to the defect state. This indicates that Na vacancies formed in Na_2_Ti_3_O_7_ accompanies with full oxidation of oxygens.

Further analysis on the partial charge density of the hole state (see the inset of Figure 5a) illustrates that, of all oxygens, one in site 5 is preferentially oxidized upon Na extraction, as predicted in Table 3. This preferential oxidation at O_5_ is also observed when Na is removed from Na_1_ (Figure S4), indicating that this phenomenon occurs regardless of the position of Na vacancy. Considering that the Schottky pairs are generated with positively charged O vacancy (*V*_O_^•^ and *V*_O_^••^, as shown in Figure 4), above hole localization behavior suggests that O vacancy in Na_2_Ti_3_O_7_ is likely to specifically form at partially oxidized O_5_. In support of this argument, we have calculated the formation energies of Schottky pairs with differing oxygen sites (Figure S5). The calculated formation energies showed that the defect pairs with O_5_ exhibit the lowest formation energy of 3.18 eV, the value of which is lower than those of other defect pairs by 0.6–2.0 eV. This confirms that, owing to the preferential oxidation of O_5_ upon Na removal, Schottky pair defects are most likely to be formed with O_5_ vacancy.

The preferential hole trapping in O_5_ can be understood by examining the local electrostatic (Madelung) potentials of oxygens in Na_2_Ti_3_O_7_. Local Madelung potential of a selected ion is determined by the sum of its electrostatic interaction with all other ions, which makes a dominant contribution to its energy position in DOS.^25,26^ When comparing the calculated Madelung potentials of oxygens (Table 4), O_5_ coordinated by single Ti has much lower value than other oxygens by more than 6.4 V. Such low electrostatic potential locates 2p orbitals of O_5_ closer to VBM compared to other O 2p orbitals. Such high-energy electrons in O_5_ tend to be readily removed upon Na extraction, resulting in the partial oxidization of O_5_.

**Table 4:** Local Bonding Environments and Madelung Potentials of All O Sites in Na_2_Ti_3_O_7_ and Na_2_Ti_6_O_13_^a^

		coordination number			average distance (Å)			
phase	O site	O–Na	O–Ti	O–O	O–Na	O–Ti	O–O	Madelung potential (V)
	O1	1	8	15	3.917	2.851	3.07	33.77
	O2	2	5	13	2.768	2.563	3.049	28.40
	O3	1	6	15	2.971	2.563	3.095	32.58
Na_2_Ti_3_O_7_	O4	3	4	12	2.528	2.83	3.222	24.49
	O5	5	1	11	2.621	1.758	3.374	17.94
	O6	3	4	13	2.696	2.811	3.256	25.28
	O7	2	6	13	2.457	3.13	3.167	25.23
								
	O1	2	6	14	2.996	3.110	3.193	23.60
	O2	2	5	13	3.456	2.548	3.018	26.71
	O3	2	4	11	2.620	2.821	3.142	25.12
Na_2_Ti_6_O_13_	O4	1	8	14	3.712	2.849	2.995	32.34
	O5	4	2	12	3.208	1.834	3.174	21.83
	O6	1	6	14	3.351	2.558	3.012	31.24
	O7	2	4	13	2.450	2.818	3.238	23.55

a Coordination numbers and average distances were determined from atoms adjacent to O by less than 4.0 Å.

The preferential oxygen vacancy formation in O_5_ as shown in Figure 5a provide insights for understanding the spontaneous phase transition of Na_2_Ti_3_O_7_ to Na_2_Ti_6_O_13_ (Figure 5b). When comparing the atomic structures of Na_2_Ti_3_O_7_ and Na_2_Ti_6_O_13_, Na_2_Ti_3_O_7_ has extra oxygen at O_5_ and two Na at Na_1_. When these O and Na atoms are removed from Na_2_Ti_3_O_7_, it can be transformed into Na_2_Ti_6_O_13_ by simple gliding of Ti–O octahedra layers along [1̅53]. This suggests that the intrinsic Schottky pairs of *V*_O_ at O_5_ and *V*_Na_ make the structure of pristine Na_2_Ti_3_O_7_ similar to Na_2_Ti_6_O_13_, facilitating the subsequent phase transition. This is especially the case when the synthesis proceeds with hydrogen sources (e.g., H_2_ gas and H_2_O vapor), which can react with Na and O of Na_2_Ti_3_O_7_ and precipitate them as Na_2_O or H_2_O.^17,27^ Under these chemical environments, the additional formation of Schottky pairs accelerates the phase transition from Na_2_Ti_3_O_7_ to Na_2_Ti_6_O_13_. The so-formed Na_2_Ti_6_O_13_ has 2D structure with relatively more interconnected Ti–O octahedra compared to Na_2_Ti_3_O_7_ and thus is more resilient to structural changes during battery cycling.

Our previous studies on the Gibbs free energies of Na_2_Ti_3_O_7_ and its competing phases showed that Na_2_Ti_3_O_7_ is a metastable phase at an operating temperature (∼300 K), whereas Na_2_Ti_6_O_13_ is thermodynamically stable at the same temperature.^16^ This indicates that once Na_2_Ti_3_O_7_ is decomposed into Na_2_Ti_6_O_13_, the reverse reaction is thermodynamically not favorable. To further investigate the reversibility of this phase transition behavior, we calculated the transition level diagrams of secondary Na_2_Ti_6_O_13_ and its dominant native defects (Figures S6 and S7). This was done for the same sets of elemental chemical potentials of A and B used in Na_2_Ti_3_O_7_ as shown in Figure 3. Overall, for both oxygen-poor and oxygen-rich conditions, Na vacancy in site 1 (*V*_Na,1_) acts as a dominant compensating acceptor defect, which has the lowest formation energy among acceptor defects for almost all Fermi energies within the band gap. The dominant donor defects in Na_2_Ti_6_O_13_ differ depending on the chemical potential conditions: oxygen vacancies (*V*_O_^•^ and *V*_O_^••^) and Ti_Na_ antisite (Ti_Na_^•••^) for oxygen-poor conditions and three defect species of Na_i_^•^, *V*_O_^••^, and Ti_Na_^•••^ for oxygen-rich conditions (Figure S6). This suggests three possible charge compensation mechanisms upon synthesis of Na_2_Ti_6_O_13_, that is, (1) Frenkel pair of *V*_Na_^′^ and Na_i_^•^, (2) Ti antisite and Na vacancy pair (Ti_Na_^•••^ and 3*V*_Na_^′^), and (3) Schottky pair of Na and O vacancies (2*V*_Na_^′^ + *V*_O_^••^ or *V*_Na_^′^ + *V*_O_^•^). To identify the dominant charge compensation mechanism, subsequent calculations were carried out on defect concentrations of Na_2_Ti_6_O_13_ at the SC Fermi level (Figure S7). The results showed that, similar to Na_2_Ti_3_O_7_, Schottky pair of Na and O vacancies act as dominant intrinsic defects, where *V*_Na_^′^ + *V*_O_^•^ pairs tend to form under oxygen-poor conditions and 2*V*_Na_^′^ + *V*_O_^••^ pairs will be created under oxygen-rich conditions. Subsequent analysis on the localization of hole/electron spin states reveals that most native defects of Na_2_Ti_6_O_13_ represent strongly localized polarons on neighboring atoms, as revealed by deep ε(0/+) and ε(0/−) presented in Table S2. In particular, Na vacancy has localized hole polaron at O_2_ with anε(0/−) value of 0.98 eV, suggesting that the positively charged oxygen vacancies (*V*_O_^•^ or *V*_O_^••^) are likely to be created at O_2_ upon the Schottky pair formation. Such Schottky defect pairs cause the atomic structure of Na_2_Ti_6_O_13_ to further deviate from Na_2_Ti_3_O_7_ (Figure 5b), which kinetically precludes Na_2_Ti_6_O_13_ from the phase transition to Na_2_Ti_3_O_7_.

Further examination on the transition level diagrams of Na_2_Ti_6_O_13_ shows that their SC Fermi levels are closer to CBM than those of Na_2_Ti_3_O_7_ by 0.05–0.12 eV (Figures S6 and 3). This suggests that Na_2_Ti_6_O_13_ is a relatively stronger n-type material with higher electron concentration, as confirmed from charge carrier concentrations calculated at the SC Fermi levels (Figure S8). These differences suggest that Na_2_Ti_6_O_13_ formed upon hydrogenation treatment has higher electrical conductivity, which improves the overall conductivity and associated rate performance of mixed titanate anodes. To investigate this behavior, we first synthesized the hybrid Na_2_Ti_3_O_7_/Na_2_Ti_6_O_13_ anode by adding 20 wt % of urea (CH_4_N_2_O) to pristine Na_2_Ti_3_O_7_, synthesized by solid-state reaction methods at 800 °C, as described in ref (16), followed by a heat treatment at 450 °C under a N_2_ atmosphere. Under the heat treatment, urea can act as a hydrogen source by a two-step decomposition process^16^
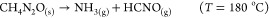
5
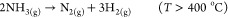
6

**Figure 6:**
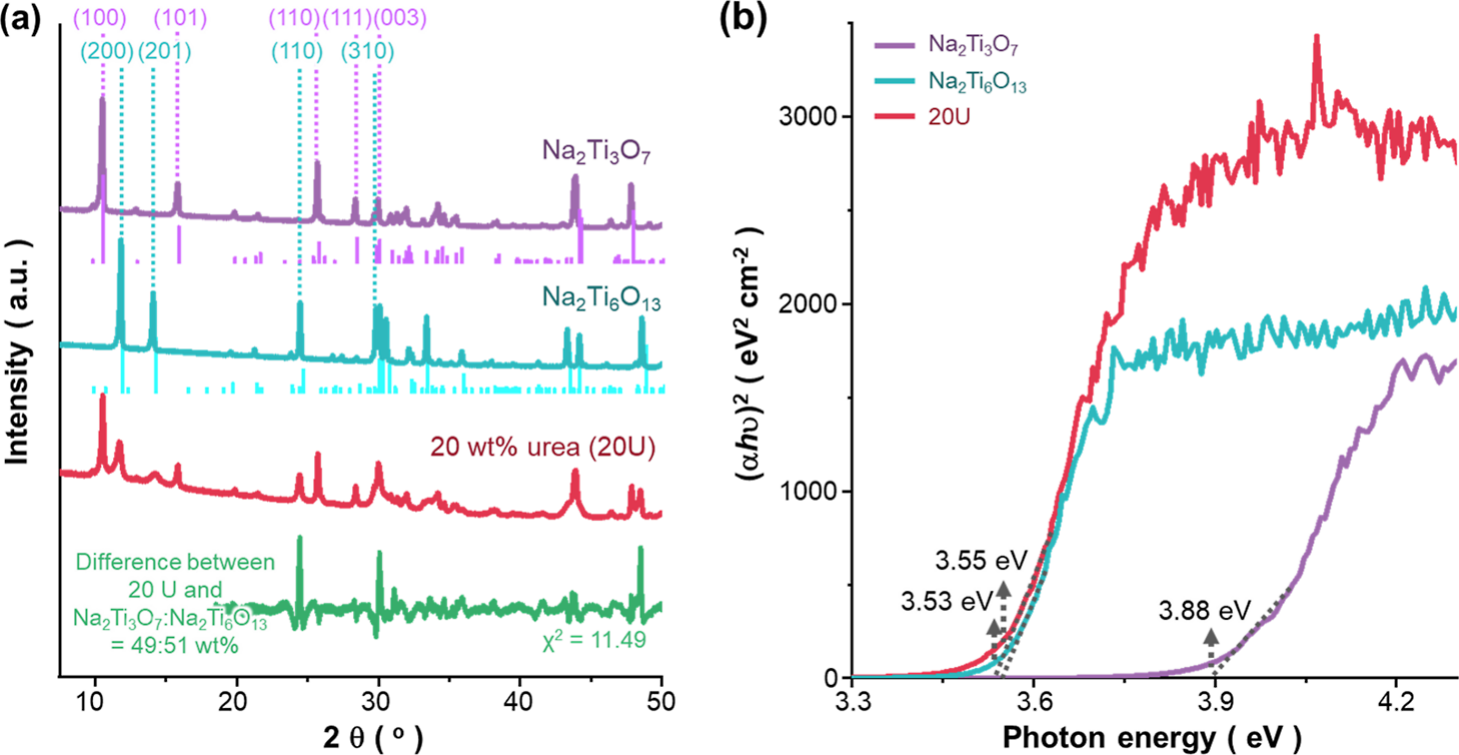
(a) PXRD data measured for Na_2_Ti_3_O_7_, Na_2_Ti_6_O_13_, and the 20 wt % urea-treated titanate anode (20U). Bragg reflections of Na_2_Ti_3_O_7_ and Na_2_Ti_6_O_13_ in low refractive index ranges in 10° < 2θ < 30° are denoted by purple and cyan tick marks, respectively, whereas XRD from calculated structures are indicated by solid vertical lines. Difference between experimental and calculated data resulting from Rietveld fit against XRD data of the 20U sample is added for reference. (b) Tauc plots obtained from the ultraviolet–visible diffusive reflectance spectra in the range of 200–600 nm measured for Na_2_Ti_3_O_7_, Na_2_Ti_6_O_13_, and the urea-treated anode. See Figures S9–S10 and Table S4 in Supporting Information for the raw data.

The urea treatment causes the phase transition of Na_2_Ti_3_O_7_ anode such that the resultant mixed titanate anode is composed of Na_2_Ti_3_O_7_ and Na_2_Ti_6_O_13_ of 49 and 51 wt %, respectively (Figure 6a). Such phase transition improves the charge transfer kinetics of the anode: subsequent UV–vis spectroscopy experiments (Figure 6b) show that the band gap of the mixed anode after hydrogenation treatment has lower band gap (3.53 eV) than the pristine one (3.88 eV), while having a similar value (3.55 eV) to Na_2_Ti_6_O_13_. Furthermore, our preliminary Mott–Schottky plots^16^ showed that the urea treatment greatly increases the charge carrier density of the anode by ∼33 times. Such improvements in charge transfer processes boost the rate performance of the anode: the 20 wt % urea sample can deliver 53% of the initial capacity (272 mA h g^–1^) after 100 cycles under a 2 C rate, whereas the pristine Na_2_Ti_3_O_7_ anode shows only 42% capacity retention compared to its initial capacity (210 mA h g^–1^) under the same condition (100 cycles at 2 C).^16^

Overall, the phase transition and associated improvements in charge transfer kinetics are more significant in experiments than calculations. For instance, the amount of Na_2_Ti_6_O_13_ formed after hydrogenation treatment (51%) is much greater than the predicted value of 7–8% (see Figure 4). In addition, compared to the calculated decrements in band gap of mixed anodes (∼0.19 eV),^16^ the measured band gap is lowered by 0.35 eV after synthesis. In the case of charge carrier concentration, it is measured to be greater for mixed anodes than pristine Na_2_Ti_3_O_7_ by 33 times,^16^ whereas the electron concentration of Na_2_Ti_6_O_13_ (1.22 × 10^18^ cm^–3^) is predicted to be greater than Na_2_Ti_3_O_7_ (4.11 × 10^16^ cm^–3^) by up to 30 times under n-type conditions at a synthesis temperature of 1170 K. The above differences between experiments and calculations are speculated to arise from the hydrogen sources that were overlooked in calculations. Although it was not considered in the present calculations, urea and its decomposed components can facilitate the removal of Na and O from Na_2_Ti_3_O_7_ and thus increase the amount of secondary Na_2_Ti_6_O_13_ after synthesis. This can be confirmed from our previous electron paramagnetic resonance spectroscopy measurements,^16^ where more oxygen vacancies and associated Ti^3+^ ions are generated with increasing urea contents. Furthermore, in addition to the intrinsic defects calculated in this study, urea treatment can create extrinsic defects including hydroxyl groups and nitrogen interstitials. These defects can further increase the charge carrier concentrations while reducing the band gap of the mixed anode, which will be identified in the future by calculating the effect of potential extrinsic defects created upon urea treatment.

One effective way to quantitatively represent the improved charge transfer kinetics of mixed anodes is to estimate their electrical conductivity using electrochemical impedance spectroscopy.^28^ Experimentally, certain requirements need to be met to allow reliable electrochemical impedance spectroscopy data analysis, for example, low porosity, as pores constrain the current flow during the experiment, resulting in Nyquist plots with large impedances, which do not reflect accurately the material’s properties.^29^ To achieve densified pellets, methods including high-temperature sintering,^30^ spark plasma sintering,^31^ and two-step sintering processes^32^ have been studied. These methods, however, can lead to element volatilization, due to localized sample overheating. For Na_2_Ti_3_O_7_, defect formation due to Na and O volatilization may trigger phase transformations to Na_2_Ti_6_O_13_, preventing from establishing an accurate relationship between a phase and its specific conductivity.

**Figure 7:**
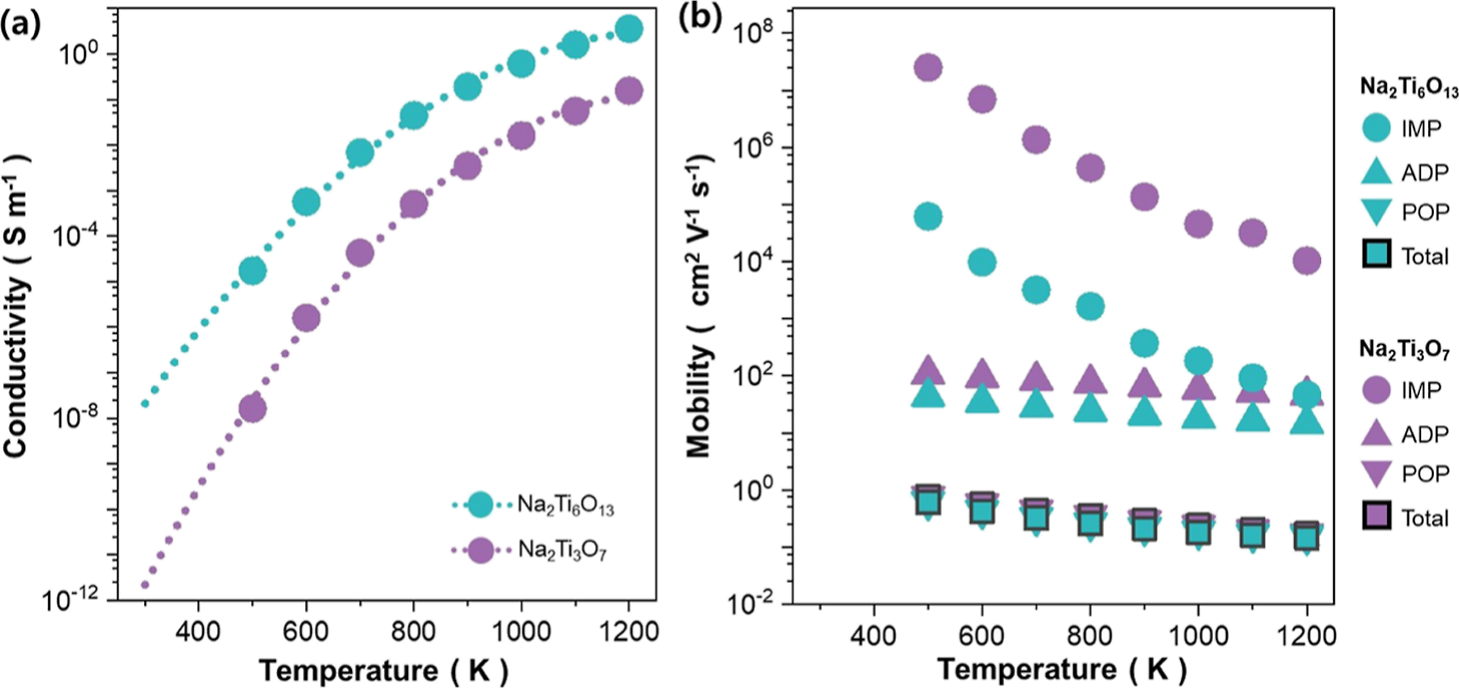
(a) Calculated electrical conductivity of Na_2_Ti_3_O_7_, Na_2_Ti_6_O_13_, and mixture of Na_2_Ti_3_O_7_ Na_2_Ti_6_O_13_ anodes. (b) Temperature-dependent theoretical mobility of Na_2_Ti_3_O_7_ and Na_2_Ti_6_O_13_ as calculated by AMSET under differing scattering mechanisms of POP, IMP, and ADP.

For this reason, we employed the ab initio scattering and transport (AMSET) software package to calculate the electrical conductivity solely from first-principles input. The AMSET calculations have shown excellent agreement against both experiments and state-of-the-art calculations on semiconducting materials.^33^ The list of parameters that have been used in AMSET for mobility calculations are provided in Table S5, where three different scattering mechanisms of polar optical phonon (POP), ionized impurity (IMP), and acoustic deformation potential (ADP) are considered under the momentum relaxation time approximation^34^ (see Methods). When calculating the electrical conductivity, charge carrier concentrations are assumed to be those at the SC Fermi level of chemical potential A (O-poor conditions) as shown in Figures 2 and S3, where sufficient amount of electron concentrations (>10^10^ cm^–3^) are predicted under temperature greater than 500 K S7. The calculated electrical conductivity in Figure 7a shows that Na_2_Ti_6_O_13_ exhibits greater conductivity than Na_2_Ti_3_O_7_ by 4 orders of magnitude at 300 K. The higher electrical conductivity of Na_2_Ti_6_O_13_ makes the hybrid titanate anodes to have enhanced charge transfer kinetics, thereby exhibiting improved rate performance as reported in the literature.^14^

To better understand the origin of the high electrical conductivity calculated in Figure 7a, we analyzed the scattering physics governing the charge transfer of Na_2_Ti_3_O_7_ and Na_2_Ti_3_O_13_. Figure 7b shows the calculated electron mobilities of Na_2_Ti_3_O_7_ and Na_2_Ti_6_O_13_ and their corresponding scattering mechanisms. For both materials, the major mechanism that limits the electron mobility is predicted to be POP, which is followed by ADP and IMP. The effect of POP scattering is more significant for Na_2_Ti_6_O_13_, which causes its electron mobility to be lower than that of Na_2_Ti_3_O_7_. Even with lower mobility, Na_2_Ti_6_O_13_ exhibits higher electrical conductivity than Na_2_Ti_3_O_7_ (Figure 7a). Considering that the electrical conductivity σ is given by σ = *nq*μ (*n*, *q*, and μ correspond to the electron density, electron charge, and the mobility, respectively), this is speculated to arise from the higher electron concentration of Na_2_Ti_6_O_13_, as shown by the calculated charge carrier densities in Figure S8.

## 3 Conclusions

In this study, by considering various ranges of native defects with differing charge states, we provided a detailed picture of defect formation mechanism of Na_2_Ti_3_O_7_: (1) among various native defects, *V*_Na_^′^ shows the highest defect concentrations in all chemical potential conditions accessible during synthesis. (2) Upon the formation of *V*_Na_^′^, electrons are preferentially extracted from oxygens (O_5_) that are coordinated by one Ti atom, oxidizing it to O_5_^–^. (3) *V*_O_^•^ and *V*_O_^••^, having second and third highest defect concentrations, tend to be created at oxidized O_5_ and paired with *V*_Na_^′^ to form Schottky pairs. The so-formed Schottky pairs act as a major charge compensation mechanism that reduces the charge carrier density of Na_2_Ti_3_O_7_, lowering the overall electrical conductivity. The main charge carriers of Na_2_Ti_3_O_7_ are electrons, which suggests that n-type doping will be suitable for improving anode performance by increasing charge carrier density and further shifting the Fermi energy to CBM. In contrast, p-type dopants and associated holes will be readily charge compensated by low formation energy donor defects, that is, O vacancies, which can distort atomic structures of anodes.

In addition to its role in charge compensation, Schottky pair defect is also speculated to be the root cause of the spontaneous phase transition from Na_2_Ti_3_O_7_ to Na_2_Ti_6_O_13_ upon hydrogenation/hydrothermal synthesis: Schottky pair defects not only change stoichiometries of Na_2_Ti_3_O_7_ close to Na_2_Ti_6_O_13_ but also cause Na_2_Ti_3_O_7_ to be readily transformed into Na_2_Ti_6_O_13_ by simple gliding of Ti–O octahedra layers. The resulting Na_2_Ti_6_O_13_ exhibits a sturdier framework of the Ti–O octahedra than Na_2_Ti_3_O_7_, which contributes to the improved cycling performance of titanate anodes after phase transition.^14,16,17^ Subsequent experiments show that urea treatment on the pristine Na_2_Ti_3_O_7_ anode can act as hydrogen sources, which accelerates the Schottky pair formations and the spontaneous phase transition from Na_2_Ti_3_O_7_ to Na_2_Ti_6_O_13_. The resulting mixture anode shows the band gap to lower by 0.35 eV, as revealed by UV–vis spectroscopic measurements. The enhanced charge transfer kinetics of the mixed anodes were further supported by our previous Mott–Schottky measurements that showed increased charge carrier concentrations for the mixed anodes.^16^ First-principles calculations together with advanced scattering modes quantitatively assessed the relatively high electrical conductivity of Na_2_Ti_6_O_13_ that contributes to the enhanced rate performance of the mixed titanate anodes. The improved electrical conductivity of Na_2_Ti_6_O_13_ mainly arises from the greater amount of electron concentration compared to Na_2_Ti_3_O_7_.

Our investigation illustrates the intrinsic defect chemistry of Na_2_Ti_3_O_7_ and its potential applications to optimize electrochemical performances. Specifically, donor dopants will increase the charge carrier density and are suitable for improving its electrical conductivity. In addition, we expect these dopants may induce the creation of the low formation energy acceptor defects of *V*_Na_, which may enable the vacancy hoping migration of Na and increase the ionic conductivity of Na_2_Ti_3_O_7_. From this perspective, n-type doping, with its capability to improve both electric and ionic conductivity, is the way to mitigate the inherent drawbacks of Na_2_Ti_3_O_7_ and fully utilize its high specific capacity under fast charging/discharging conditions. Another effective strategy is to supply the reducing agents during synthesis to induce the formation of Na_2_Ti_6_O_13_ with a sturdier 3D tunnel Ti–O framework. We have been demonstrated this argument using one of the affordable reducing agents, urea, where the content of Na_2_Ti_6_O_13_ increases in the titanate anodes with increasing amount of urea.^16^ The resulting mixture titanate anodes will be characterized by stable cyclability at the expense of low specific capacity,^14^ both features of which correspond to tunnel-structured Na_2_Ti_6_O_13_. As this phenomenon is the same for another reducing agent of H_2_ gas,^27^ we expect various other additives (e.g., sodium borohydride, hydrazine hydrate) can perform the same roles. The above strategies provide directions to optimize Na_2_Ti_3_O_7_ anode either for developing large energy storage devices with high specific capacity or for fast chargeable anodes with long cycle life.

## Supporting Information Available

Methods; thermodynamic stability of phases in the Na–Ti–O system; comparison on the entropy values of elemental phases of Na and Ti; chemical potential stability region of Na_2_Ti_6_O_13_; DOS of Na_2_Ti_3_O_7_ after the removal of a Na atom; Schottky defect pairs in Na_2_Ti_3_O_7_ and their formation energies; transition level diagram of Na_2_Ti_6_O_13_; equilibrium defect concentrations of Na_2_Ti_6_O_13_; charge carrier densities of Na_2_Ti_3_O_7_ and Na_2_Ti_6_O_13_; Rietveld refinements of titanate samples; UV–vis spectroscopy data of titanate samples; lattice parameters of Na_2_Ti_3_O_7_ and Na_2_Ti_6_O_13_; thermodynamic transition levels of native defects in Na_2_Ti_6_O_13_; list of localized of polarons of Na_2_Ti_6_O_13_; crystallographic data of Na_2_Ti_3_O_7_ and Na_2_Ti_6_O_13_ from Rietveld refinements; input parameters to AMSET; and additional notes regarding the number of mobile Na ions of Na_2_Ti_3_O_7_ (PDF)

## References

[ref1] Tapia-Ruiz N.; Armstrong A. R.; Alptekin H.; Amores M. A.; Au H.; Barker J.; Boston R.; Brant W. R.; Brittain J. M.; Chen Y.; Chhowalla M.; Choi Y.-S.; Costa S. I. R.; Crespo Ribadeneyra M. C.; Cussen S. A.; Cussen E. J.; David W. I. F.; Desai A. V.; Dickson S. A. M.; Eweka E. I.; Forero-Saboya J. D.; Grey C. P.; Griffin J. M.; Gross P.; Hua X.; Irvine J. T. S.; Johansson P.; Jones M. O.; Karlsmo M.; Kendrick E.; Kim E.; Kolosov O. V.; Li Z.; Mertens S. F. L.; Mogensen R.; Monconduit L.; Morris R. E.; Naylor A. J.; Nikman S.; O’Keefe C. A.; Ould D. M. C.; Palgrave R. G.; Poizot P.; Ponrouch A.; Renault S.; Reynolds E. M.; Rudola A.; Sayers R.; Scanlon D. O.; Sen S.; Seymour V. R.; Silván B.; Sougrati M. T.; Stievano L.; Stone G. S.; Thomas C. I.; Titirici M.-M.; Tong J.; Wood T. J.; Wright D. S.; Younesi R. 2021 roadmap for sodium-ion batteries. J. Phys.: Energy 2021, 3, 031503 10.1088/2515-7655/ac01ef.

[ref2] Booth S. G.; Nedoma A. J.; Anthonisamy N. N.; Baker P. J.; Boston R.; Bronstein H.; Clarke S. J.; Cussen E. J.; Daramalla V.; De Volder M.; Dutton S. E.; Falkowski V.; Fleck N. A.; Geddes H. S.; Gollapally N.; Goodwin A. L.; Griffin J. M.; Haworth A. R.; Hayward M. A.; Hull S.; Inkson B. J.; Johnston B. J.; Lu Z.; MacManus-Driscoll J. L.; Martínez De Irujo Labalde X.; McClelland I.; McCombie K.; Murdock B.; Nayak D.; Park S.; Pérez G. E.; Pickard C. J.; Piper L. F. J.; Playford H. Y.; Price S.; Scanlon D. O.; Stallard J. C.; Tapia-Ruiz N.; West A. R.; Wheatcroft L.; Wilson M.; Zhang L.; Zhi X.; Zhu B.; Cussen S. A. Perspectives for next generation lithium-ion battery cathode materials. APL Mater. 2021, 9, 109201 10.1063/5.0051092.

[ref3] Dong S.; Shen L.; Li H.; Pang G.; Dou H.; Zhang X. Flexible sodium-ion pseudocapacitors based on 3D Na2Ti3O7 nanosheet arrays/carbon textiles anodes. Adv. Funct. Mater. 2016, 26, 3703–3710. 10.1002/adfm.201600264.

[ref4] Liao J.-Y.; Manthiram A. High-performance Na2Ti2O5 nanowire arrays coated with VS2 nanosheets for sodium-ion storage. Nano Energy 2015, 18, 20–27. 10.1016/j.nanoen.2015.09.014.

[ref5] Senguttuvan P.; Rousse G.; Seznec V.; Tarascon J.-M.; Palacín M. R. Na2Ti3O7: lowest voltage ever reported oxide insertion electrode for sodium ion batteries. Chem. Mater. 2011, 23, 4109–4111. 10.1021/cm202076g.

[ref6] Xu J.; Ma C.; Balasubramanian M.; Meng Y. S. Understanding Na 2 Ti 3 O 7 as an ultra-low voltage anode material for a Na-ion battery. Chem. Commun. 2014, 50, 12564–12567. 10.1039/c4cc03973d. 25198509

[ref7] Rudola A.; Sharma N.; Balaya P. Introducing a 0.2 V sodium-ion battery anode: The Na2Ti3O7 to Na3– xTi3O7 pathway. Electrochem. Commun. 2015, 61, 10–13. 10.1016/j.elecom.2015.09.016.

[ref8] Dong S.; Shen L.; Li H.; Nie P.; Zhu Y.; Sheng Q.; Zhang X. Pseudocapacitive behaviours of Na 2 Ti 3 O 7@ CNT coaxial nanocables for high-performance sodium-ion capacitors. J. Mater. Chem. A 2015, 3, 21277–21283. 10.1039/c5ta05714k.

[ref9] Yan Z.; Liu L.; Shu H.; Yang X.; Wang H.; Tan J.; Zhou Q.; Huang Z.; Wang X. A tightly integrated sodium titanate-carbon composite as an anode material for rechargeable sodium ion batteries. J. Power Sources 2015, 274, 8–14. 10.1016/j.jpowsour.2014.10.045.

[ref10] Zarrabeitia M.; Castillo-Martínez E.; López Del Amo J. M. L.; Eguía-Barrio A.; Muñoz-Márquez M. Á.; Rojo T.; Casas-Cabanas M. Identification of the critical synthesis parameters for enhanced cycling stability of Na-ion anode material Na2Ti3O7. Acta Mater. 2016, 104, 125–130. 10.1016/j.actamat.2015.11.033.

[ref11] Chen J.; Zhou X.; Mei C.; Xu J.; Wong C.-P. Improving the sodiation performance of Na2Ti3O7 through Nb-doping. Electrochim. Acta 2017, 224, 446–451. 10.1016/j.electacta.2016.12.094.

[ref12] Xia J.; Zhao H.; Pang W. K.; Yin Z.; Zhou B.; He G.; Guo Z.; Du Y. Lanthanide doping induced electrochemical enhancement of Na 2 Ti 3 O 7 anodes for sodium-ion batteries. Chem. Sci. 2018, 9, 3421–3425. 10.1039/c7sc05185a. 29844899 PMC5931090

[ref13] Chen Z.; Lu L.; Gao Y.; Zhang Q.; Zhang C.; Sun C.; Chen X. Effects of F-doping on the electrochemical performance of Na2Ti3O7 as an anode for sodium-ion batteries. Materials 2018, 11, 2206 10.3390/ma11112206. 30405040 PMC6266345

[ref14] Wu C.; Hua W.; Zhang Z.; Zhong B.; Yang Z.; Feng G.; Xiang W.; Wu Z.; Guo X. Design and synthesis of layered Na2Ti3O7 and tunnel Na2Ti6O13 hybrid structures with enhanced electrochemical behavior for sodium-ion batteries. Adv. Sci. 2018, 5, 1800519 10.1002/advs.201800519. PMC6145307 30250795

[ref15] Hwang J.; Setiadi Cahyadi H. S.; Chang W.; Kim J. Uniform and ultrathin carbon-layer coated layered Na2Ti3O7 and tunnel Na2Ti6O13 hybrid with enhanced electrochemical performance for anodes in sodium ion batteries. J. Supercrit. Fluids 2019, 148, 116–129. 10.1016/j.supflu.2019.03.006.

[ref16] Costa S. I.; Choi Y. S.; Fielding A. J.; Naylor A. J.; Griffin J. M.; Sofer Z.; Scanlon D. O.; Tapia-Ruiz N. Surface Engineering Strategy Using Urea To Improve the Rate Performance of Na2Ti3O7 in Na-Ion Batteries. Chem.—Eur. J. 2021, 27, 3875–3886. 10.1002/chem.202003129. 32852862 PMC7986851

[ref17] Liu H.; Yang D.; Zheng Z.; Ke X.; Waclawik E.; Zhu H.; Frost R. L. A Raman spectroscopic and TEM study on the structural evolution of Na2Ti3O7 during the transition to Na2Ti6O13. J. Raman Spectrosc. 2010, 41, 1331–1337. 10.1002/jrs.2561.

[ref18] Krukau A. V.; Vydrov O. A.; Izmaylov A. F.; Scuseria G. E. Influence of the exchange screening parameter on the performance of screened hybrid functionals. J. Chem. Phys. 2006, 125, 224106 10.1063/1.2404663. 17176133

[ref19] Perdew J. P.; Ruzsinszky A.; Csonka G. I.; Vydrov O. A.; Scuseria G. E.; Constantin L. A.; Zhou X.; Burke K. Restoring the density-gradient expansion for exchange in solids and surfaces. Phys. Rev. Lett. 2008, 100, 136406 10.1103/physrevlett.100.136406. 18517979

[ref20] Chen S.; Gong X.; Walsh A.; Wei S.-H. Defect physics of the kesterite thin-film solar cell absorber Cu 2 ZnSnS 4. Appl. Phys. Lett. 2010, 96, 021902 10.1063/1.3275796.

[ref21] Buckeridge J.; Scanlon D. O.; Walsh A.; Catlow C. R. A. Automated procedure to determine the thermodynamic stability of a material and the range of chemical potentials necessary for its formation relative to competing phases and compounds. Comput. Phys. Commun. 2014, 185, 330–338. 10.1016/j.cpc.2013.08.026.

[ref22] Tygesen A. S.; Chang J. H.; Vegge T.; García-Lastra J. M. Computational framework for a systematic investigation of anionic redox process in Li-rich compounds. npj Comput. Mater. 2020, 6, 65 10.1038/s41524-020-0335-4.

[ref23] Chang J. H.; Baur C.; Ateba Mba J.-M. A.; Arčon D.; Mali G.; Alwast D.; Behm R. J.; Fichtner M.; Vegge T.; Garcia Lastra J. M. G. Superoxide formation in Li 2 VO 2 F cathode material–a combined computational and experimental investigation of anionic redox activity. J. Mater. Chem. A 2020, 8, 16551–16559. 10.1039/d0ta06119k.

[ref24] Van de Walle C. G.; Neugebauer J. First-principles calculations for defects and impurities: Applications to III-nitrides. J. Appl. Phys. 2004, 95, 3851–3879. 10.1063/1.1682673.

[ref25] Scanlon D. O.; Dunnill C. W.; Buckeridge J.; Shevlin S. A.; Logsdail A. J.; Woodley S. M.; Catlow C. R. A.; Powell M.; Palgrave R. G.; Parkin I. P.; Watson G. W.; Keal T. W.; Sherwood P.; Walsh A.; Sokol A. A. Band alignment of rutile and anatase TiO2. Nat. Mater. 2013, 12, 798–801. 10.1038/nmat3697. 23832124

[ref26] Buckeridge J.; Butler K. T.; Catlow C. R. A.; Logsdail A. J.; Scanlon D. O.; Shevlin S. A.; Woodley S. M.; Sokol A. A.; Walsh A. Polymorph engineering of TiO2: demonstrating how absolute reference potentials are determined by local coordination. Chem. Mater. 2015, 27, 3844–3851. 10.1021/acs.chemmater.5b00230.

[ref27] Kolen’ko Y. V.; Kovnir K. A.; Gavrilov A. I.; Garshev A. V.; Frantti J.; Lebedev O. I.; Churagulov B. R.; Van Tendeloo G.; Yoshimura M. Hydrothermal synthesis and characterization of nanorods of various titanates and titanium dioxide. J. Phys. Chem. B 2006, 110, 4030–4038. 10.1021/jp055687u. 16509693

[ref28] Irvine J. T.; Sinclair D. C.; West A. R. Electroceramics: characterization by impedance spectroscopy. Adv. Mater. 1990, 2, 132–138. 10.1002/adma.19900020304.

[ref29] Cordier A.; El Khal H.; Siebert E.; Steil M. C. On the role of the pore morphology on the electrical conductivity of porous yttria-stabilized zirconia. J. Eur. Ceram. Soc. 2019, 39, 2518–2525. 10.1016/j.jeurceramsoc.2019.02.027.

[ref30] Tong J.; Clark D.; Bernau L.; Sanders M.; O’Hayre R. Solid-state reactive sintering mechanism for large-grained yttrium-doped barium zirconate proton conducting ceramics. J. Mater. Chem. 2010, 20, 6333–6341. 10.1039/c0jm00381f.

[ref31] Manière C.; Lee G.; Olevsky E. A. All-materials-inclusive flash spark plasma sintering. Sci. Rep. 2017, 7, 15071 10.1038/s41598-017-15365-x. 29118370 PMC5678114

[ref32] Chinelatto A. S. A.; Manosso M. K.; Pallone E. M. J. A.; Souza A. M.; Chinelatto A. L. Effect of the two-step sintering in the microstructure of ultrafine alumina. Adv. Sci. Technol. 2010, 62, 221–226. 10.4028/www.scientific.net/ast.62.221.

[ref33] Pöhls J.-H.; Chanakian S.; Park J.; Ganose A. M.; Dunn A.; Friesen N.; Bhattacharya A.; Hogan B.; Bux S.; Jain A.; Mar A.; Zevalkink A. Experimental validation of high thermoelectric performance in RECuZnP 2 predicted by high-throughput DFT calculations. Mater. Horiz. 2021, 8, 209–215. 10.1039/d0mh01112f. 34821299

[ref34] Poncé S.; Li W.; Reichardt S.; Giustino F. First-principles calculations of charge carrier mobility and conductivity in bulk semiconductors and two-dimensional materials. Rep. Prog. Phys. 2020, 83, 036501 10.1088/1361-6633/ab6a43. 31923906

