# Supplementary material for: Intrinsic Defects and Their Role in the Phase Transition of Na-Ion Anode Na2Ti3O7
Source: ACS Appl Energy Mater. 2022 Dec 16;6(1):484–95. doi: 10.1021/acsaem.2c03466 (PMC9832431; doi:10.1021/acsaem.2c03466)
Supplement: Supplementary file 1 — ae2c03466_si_001.pdf [file ae2c03466_si_001.pdf]

Supporting Information

Intrinsic defects and their role in the phase transition of Na-ion anode $Na_2Ti_3O_7$

Yong-Seok Choi,[1,2] Sara I. R. Costa,[3,5] Nuria Tapia-Ruiz,[3,5] and David O. Scanlon*[2,3,4]

[1]Department of Materials Science and Engineering, Dankook University, Cheonan 31116, South Korea

[2]Department of Chemistry, University College London, 20 Gordon Street, London WC1H 0AJ, UK

[3]The Faraday Institution, Harwell Campus, Didcot OX11 0RA, UK

[4]Thomas Young Centre, University College London, Gower Street, London WC1E 6BT, UK

[5]Department of Chemistry, Lancaster University, Lancaster LA1 4YB, UK

E-mail: d.scanlon@ucl.ac.uk

Table of Contents

Methods 2

List of Figures

Figure S1. Thermodynamic stability of phases in Na-Ti-O system 6
Figure S2. Comparison on the entropy values of elemental phases of Na and Ti 6
Figure S3. Chemical potential stability region of $Na_2Ti_6O_{13}$ 7
Figure S4. Density of states of $Na_2Ti_3O_7$ after the removal of a Na atom 7
Figure S5. Schottky defect pairs in $Na_2Ti_3O_7$ and their formation energies 8
Figure S6. Transition-level diagram of $Na_2Ti_6O_{13}$ 8
Figure S7. Equilibrium defect concentrations of $Na_2Ti_6O_{13}$ 9
Figure S8. Charge carrier densities of $Na_2Ti_3O_7$ and $Na_2Ti_6O_{13}$ 9
Figure S9. Rietveld refinements of titanate samples 10
Figure S10. UV-vis spectroscopy data of titanate samples 11

List of Tables

Table S1. Lattice parameters of $Na_2Ti_3O_7$ and $Na_2Ti_6O_{13}$ 12
Table S2. Thermodynamic transition levels of native defects in $Na_2Ti_6O_{13}$ 13
Table S3. List of localized of polarons of $Na_2Ti_6O_{13}$ 14
Table S4. Crystallographic data of $Na_2Ti_3O_7$ and $Na_2Ti_6O_{13}$ from Rietveld refinements 15
Table S5. Input parameters to AMSET 16

Additional Notes 17

References 19

Methods

Synthesis and physicochemical characterization

$Na_2Ti_3O_7$ and $Na_2Ti_3O_7/Na_2Ti_6O_{13}$ composite synthesized with 20wt% urea were prepared as detailed in Ref. [1]. $Na_2Ti_6O_{13}$ was prepared using an identical synthetic route to $Na_2Ti_3O_7$ but precursors were calcined at 900 °C for 25 h.

Samples were characterised by powder X-ray diffraction (PXRD) at room temperature, using a Smartlab diffractometer (Rigaku Corporation) equipped with a 9 kW Cu rotating anode (λ = 1.54056 Å) operating in reflection mode with Bragg-Brentano geometry. Data were collected in the 5-70 ° 2θ range at a scan speed of 0.02 ° s^{-1}. Rietveld refinement of the $Na_2Ti_3O_7/Na_2Ti_6O_{13}$ composite sample was performed from X-ray diffraction data, using the ICSD 250000 ICSD 23877 $Na_2Ti_3O_7$ and $Na_2Ti_6O_{13}$ structures as initial models. The GSAS-EXPGUI software interface[2, 3] was used to perform the Rietveld refinement. Peak shapes were modelled with a Gaussian-Lorentzian function, and the background, lattice parameters, atomic positions and thermal parameters were refined. The occupancy for all atoms was fixed to n = 1. For the structural refinement of the $Na_2Ti_3O_7/Na_2Ti_6O_{13}$ composite, UISO values for identical atoms were constrained to be equal.

UV-visible spectroscopy was performed to determine the bandgap of the three titanate samples described in this work using a Cary500 spectrometer in the 250-600 nm, coupled with an integrating sphere, to acquire only the diffusive reflectance of the electromagnetic radiation.

Ab initio calculations

All the calculations performed in this work employed density functional theory (DFT) as implemented in the Vienna Ab initio Simulation Package code.[4, 5] Interactions between core and valence electrons were described using the projector augmented wave (PAW) method.[6] The electron configurations Na ($3s^1$), Ti ($3d^34s^1$), and O ($2s^22p^4$) were treated as the valence electrons. Convergence to plane wave energy was checked, with a cut-off of 500 eV found to be sufficient to converge the total energy to within 0.01 eV $atom^{-1}$. Brillouin zones for all compounds were sampled such that the k-points were converged in an accuracy of the total energy in 0.001 eV $atom^{-1}$. All calculations were deemed to be converged when the forceson all atoms were less than 0.01 eV $Å^{-1}$.

To accurately predict the phase stability of $Na_2Ti_3O_7$ and $Na_2Ti_6O_{13}$, we used hybrid functional to recalculate enthalpies of phases of the Na-Ti-O system that are previously investigated with PBEsol functional.[1] In this calculation, the screened hybrid functional (HSE06),[7] in which 25% of exact non-local Fock exchange is added to the PBE[8] functional, is selected to investigate phase stability of 20 different phases of the Na-Ti-O system that are reported to be

stable in the Materials Project database (Figure S1a).[9] To take into account the temperature effect on the thermodynamic stability of phases, we employed vibrational entropies of nine phases (Na, Ti, $Na_2Ti_3O_7$, $Na_2Ti_6O_{13}$, Na_2O, Na_4TiO_4, $Na_8Ti_5O_{14}$, $Na_4Ti_5O_{12}$, and TiO_2) that were calculated in our previous study.[1] For the gas phase of O_2, the empirical gas phase thermodynamic data from the NIST data [10] are utilized instead to evaluate the temperature dependence of the gas free energies. In addition to these phases, we also calculated vibrational entropies of two phases of $NaTi_2O_4$, $NaTi_5O_{10}$ that have chemical compositions close to $Na_2Ti_3O_7$ and $Na_2Ti_6O_{13}$ to better predict the chemical potential region of stability for $Na_2Ti_3O_7$ and $Na_2Ti_6O_{13}$. This was achieved by generating 3×6×2 and 2×2×2 supercells of $NaTi_2O_4$ and $NaTi_5O_{10}$, respectively, followed by calculating the harmonic force constants from atomic displacements of supercells using quasi-harmonic equations as implemented in Phonopy package.[11] However, the calculation of vibrational entropies for these large supercells is challenging with computationally intensive HSE06 functional. Furthermore, preliminary calculations revealed that HSE06 functional can largely overestimate the vibrational entropy of phases (Figure S2). For this reason, we selected PBEsol functional to calculate vibrational entropies, for it has been proven to accurately reproduce lattice parameters and lattice dynamics in solid systems while maintaining a relatively low computational cost.[12, 13] Based on the calculated enthalpy (H) and entropy (S) of phases, Gibbs free energies were then calculated as:

$$G_{Na_xTi_yO_z} = H_{Na_xTi_yO_z} - TS_{Na_xTi_yO_z} \qquad (1)$$

where T is absolute temperature. The free energy of formation for $Na_xTi_yO_z$ can then be estimated according to:

$$\Delta G_{Na_xTi_yO_z} = G_{Na_xTi_yO_z} - xG_{Na} - yG_{Ti} - zG_O \qquad (2)$$

Calculated free energies of formation were used to predict the chemical potential spaces that can be accessible during synthesis condition. This was done by comparing free energies offormation at a synthesis temperature of 1070 K,[1] and calculating the thermodynamic stability regime of $Na_2Ti_3O_7$ and $Na_2Ti_6O_{13}$ using the CPLAP code.[14]

To obtain defect formation energies, we first selected the HSE06-optimized primitive $Na_2Ti_3O_7$ and $Na_2Ti_6O_{13}$ from phase stability calculations. To minimize the interatomic interactions of a point defect with its corresponding periodic images, we then expanded the selected primitive $Na_2Ti_3O_7$ and $Na_2Ti_6O_{13}$ structures into 3×3×3 and 2×4×2 supercells, respectively. For the obtained supercells, seven different types of intrinsic defects (V_{Na}, V_O, V_{Ti}, Na_{Ti}, Na_i, O_i, and Ti_{Na}) were created in symmetry unique defect sites of $Na_2Ti_3O_7$ and $Na_2Ti_6O_{13}$ using Python packages of pymatgen[15] and bsym[16] (Table 1). All constructed defective cells were geometrically optimized using HSE06 functional, where integrations over the Brillouin zone

were carried out using the k-grids of 2×2×2.

Based on the predicted chemical potential regions and defect formation energies, the formation energy of a defect D in the charge state q at 0 K can be calculated according to:[17]

$$\Delta H_f^{D,q} = \left(E^{D,q} - E^{H}\right) + \sum_i n_i\left(E_i^o + \Delta\mu_i\right) + q\left(E_f + \varepsilon_v^H\right) + E_{corr} \quad (3)$$

where $E^{D,q}$ is the energy of the defective supercell and E^H is the energy of the host supercell.[17, 18] The second term represents the energy change during removal/addition of defect atoms, i, where n_i corresponds to the number of atoms exchanged, E_i^o is the reference energy of a defect atom, and $\Delta\mu_i$ indicates the chemical potential of the defect atom. In the third term, E_f is the Fermi level and ε_v^H represents the energy needed to add or remove an electron from the VBM to the Fermi level. E_{corr} is a correction term to account for the artificial electrostatic interaction due to periodic boundary conditions.[19, 20] The calculation of defect formation energy at elevated temperature was then carried out by including the entropy contribution for all DFT total energy terms in the conventional 0 K defect formation energy equation as follows:[21]

$$\Delta G_f^{D,q} = \left(E^{D,q} + G^{D,q} - E^{H} - G^{H}\right) + \sum_i n_i\left(E_i^o + G_i^o + \Delta\mu_i^T\right) + q\left(E_f + \varepsilon_v^H\right) + E_{corr} \quad (4)$$

Eq. (4) is the modified formula from eq. (3) in the original Supporting Information where the free energy contributions $G\ (= TS)$ are added for defective supercell ($G^{D,q}$), host supercell (G^H), and elemental phases (G_i^o) (see Supporting Information for the definition of other terms). Elemental chemical potentials $\Delta\mu_i^T$ also differs from ones evaluated at 0 K ($\Delta\mu_i$) because of the changes in the chemical potential stability region at elevated temperature. Among the free energy contributions in eq. (4), we assumed the difference in vibrational entropy arising from a point defect in a supercell structure, i.e., $G^{D,q} - G^H$, to be zero, considering that the entropy changes for adsorption and desorption of a single atom is negligible.[22, 23]. The other free energy terms from elemental and competing phases were calculated using quasi-harmonic approximation as implemented in Phonopy,[11] except O_2 gas of which entropy was obtained from NIST thermodynamic database.[10]

The thermodynamic transition level, $\varepsilon(D,q/q')$, which represents the defect formation energies at which a defect with differing charge states, (q, q'), have the same formation energy was predicted according to:

$$\varepsilon(D,q/q') = (\Delta H_f^{D,q} - \Delta H_f^{D,q'})/(q' - q) \quad (4)$$

Plotting thermodynamic transition level of all defects and their formation energy along Fermi energy, gives transition level diagram, which conveys overall defect chemistry of materials. For a given temperature, defect and charge carrier concentrations are given as a function of Fermi energy and defect formation energy and thus, varies towards the x-axis of transition level diagram. Thus, by calculating charge carrier concentrations for all defects along Fermi energy, one can predict the SC Fermi level in the middle of band gap where overall charge carriers satisfy the charge

neutrality condition, which was done in this study using the SC-FERMI code.[24] In this calculation, defect concentrations at the SC Fermi level were calculated for two selected chemical potential sets of A and B in Figure 1a at synthesis temperature of 1070 K.

The temperature dependent electrical conductivity of $Na_2Ti_3O_7$ and $Na_2Ti_6O_{13}$ were calculated using the AMSET package.[25] Instead of assuming variable or constant relaxation time, AMSET explicitly solves Boltzmann transport equation by taking account of various scattering mechanisms. This enables the exploration of scattering mechanism that limits the electron mobility of phases. In this calculation, we considered four different scattering mechanisms arising from acoustic deformation potential (ADP), piezoelectric (PIE), polar optical phonon (POP), and ionized impurity (IMP). Consideration of above scattering mechanism requires five material properties, e.g. elastic constant, piezoelectric constant, polar optical phonon frequency, high frequency dielectric constant, and static dielectric constant, which were fully determined by DFT and density functional perturbation theory (DFPT).[26, 27] In addition to above properties, the absolute deformation potentials of $Na_2Ti_3O_7$ and $Na_2Ti_6O_{13}$ were calculated by estimating the changes in energy of conduction and valence bands with respect to contraction and expansion of unit cell volumes.[25, 28] Transport properties predicted from AMSET are given as a function of temperature and charge carrier density. Among all range of temperature and carrier density, we selected ones predicted at SC Fermi level in Figure 3. The list of parameters used in AMSET for electrical conductivity calculations are summarized in Table S5.

List of figures

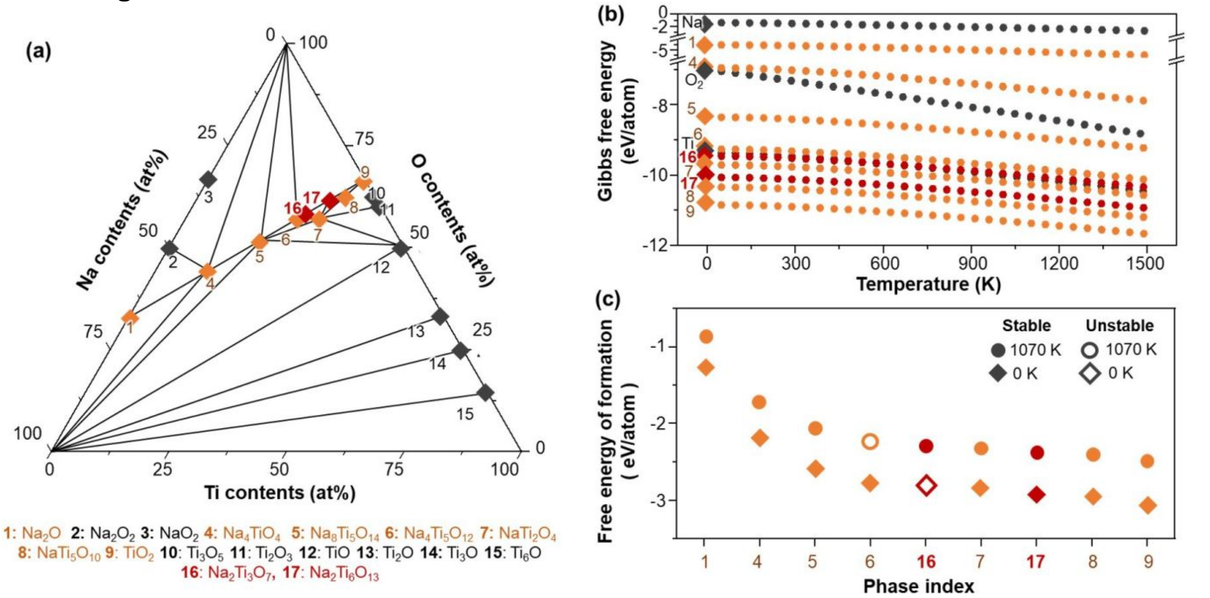

Figure S1. (a) Na-Ti-O phase diagram defined by Na, Ti and O obtained from structures with the lowest formation enthalpy calculated using HSE06 functional. Target phases of $Na_2Ti_3O_7$ and $Na_2Ti_6O_{13}$ are highlighted by red rhombus, whereas competing phases with compositions close to target phases are selected for high temperature stability and denoted by orange rhombus. Among 17 phases denoted in (a), $Na_2Ti_3O_7$ is found to be unstable at athermal limit. (b) Temperature dependence of Gibbs free energies calculated for elemental (Na, Ti, O) and selected competing phases in (a). (c) Free energies of formation calculated at 0 and 1070 K using the Gibbs free energies in (b), showing the thermodynamic stability of phases. Note that $Na_2Ti_3O_7$ is stable only at elevated temperature.

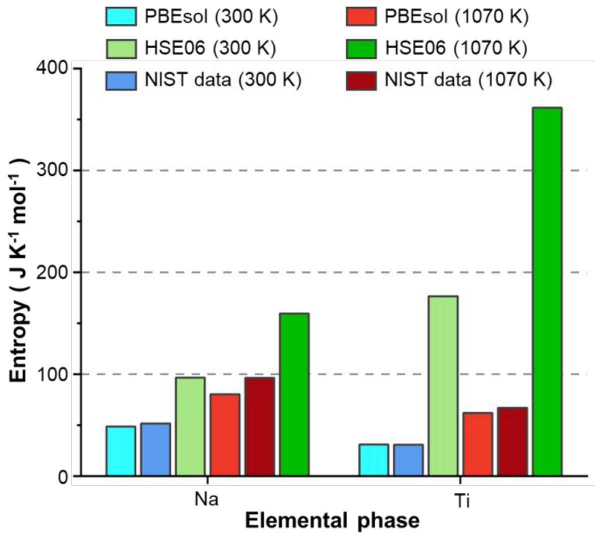

Figure S2. Comparison on the entropy values of elemental phases of Na and Ti calculated using quasi-harmonic approximation and reported in NIST thermodynamic database.[10] Note that PBEsol functionals yield the results closer to experimental values than HSE06 ones.

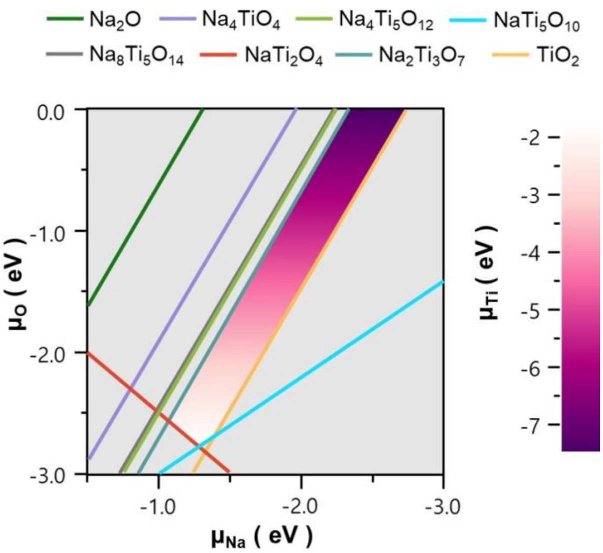

Figure S3. Region of stability (purple area) for $Na_2Ti_6O_{13}$ in the 2D space spanned by μ_{Na} and μ_O, where the variation of μ_{Ti} is shown within the stability region. The (colored) lines indicate the limits imposed by competing phases.

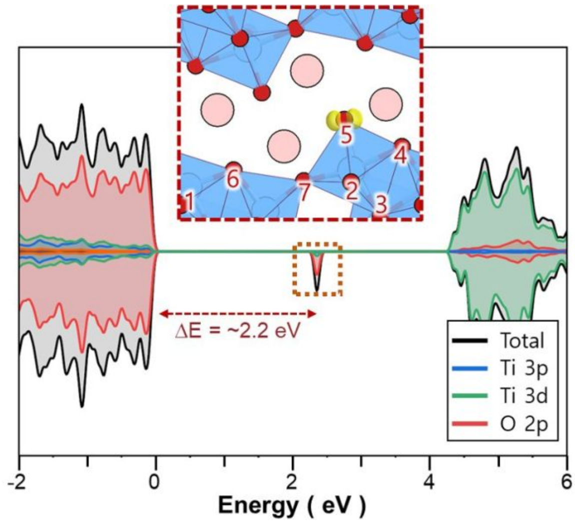

Figure S4. Projected density of states calculated for $Na_2Ti_3O_7$ after the removal of a Na atom from Na_1. The inset shows the partial charge density for the hole band located 2.2 eV above the VBM. Type of oxygen sites are denoted as numbers in the inset.

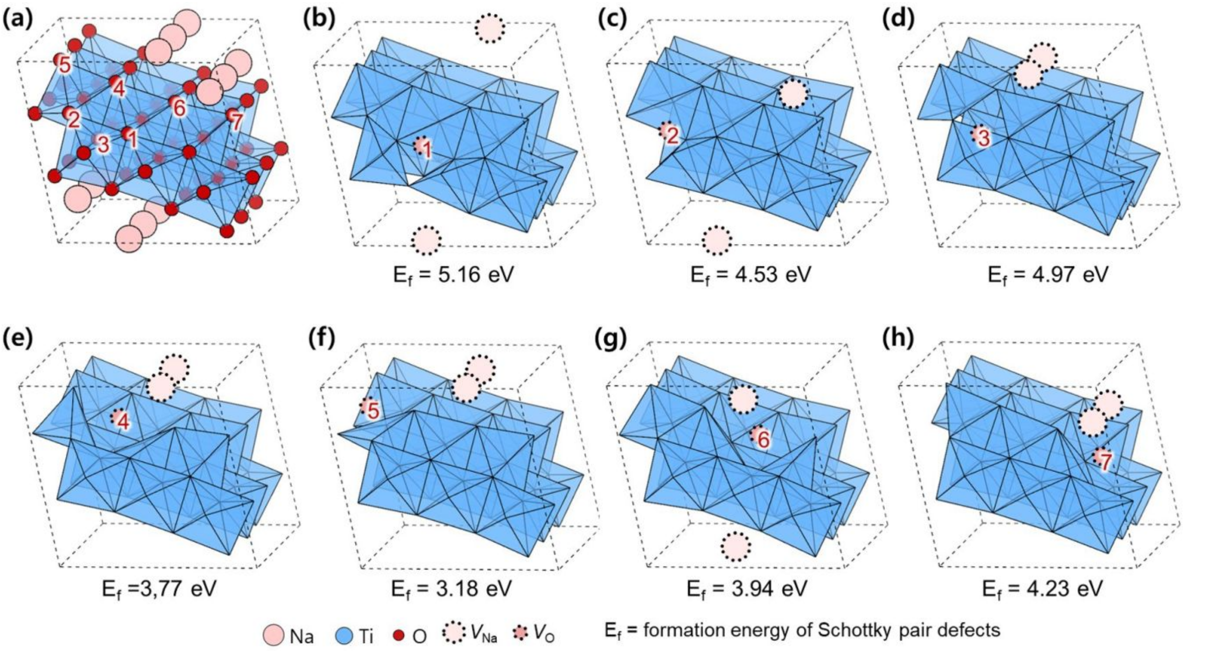

Figure S5. (a) Host crystal structures of $Na_2Ti_3O_7$ ($P2_1/m$) used for defect calculations, where symmetry unique O sites are indicated by differing numbers. (b-h) Optimized positions of Schottky defect pairs predicted when O vacancies are located at (b) O_1, (c) O_2, (d) O_3, O_4, (f) O_5, (g) O_6, and (h) O_7. The formation energy of each Schottky pair is calculated under chemical potential A of Figure 1b and denoted in (b-h). For better visualization, only vacancies and Ti-O frameworks are shown in (b-h).

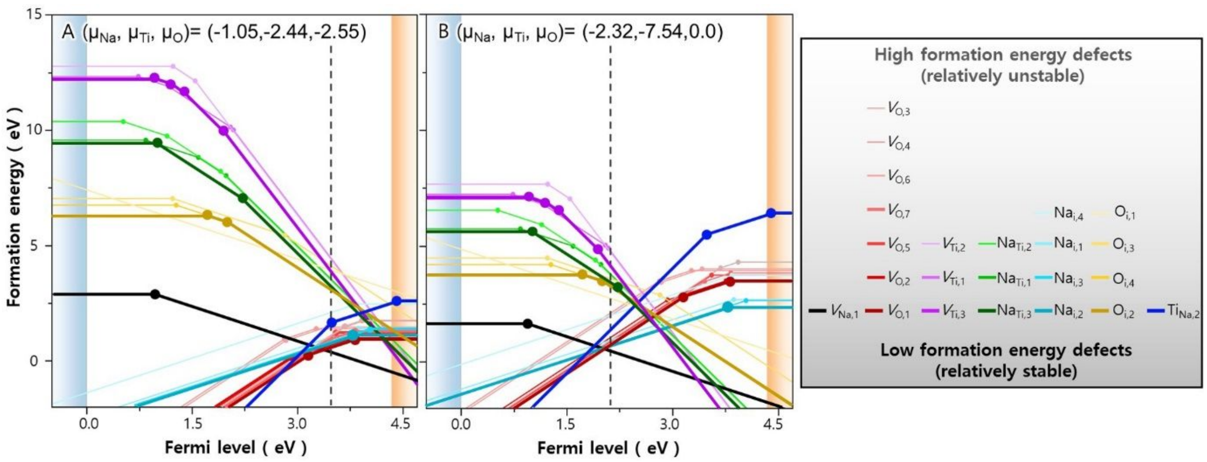

Figure S6. Transition-level diagram of $Na_2Ti_6O_{13}$ plotted under two different sets of elemental chemical potentials selected in Figure 1b, showing defect formation energies as a function of Fermi energy. Vertical dashed line denotes SC Fermi energy at 1070 K where the concentration of excess electrons and holes satisfy charge neutrality.

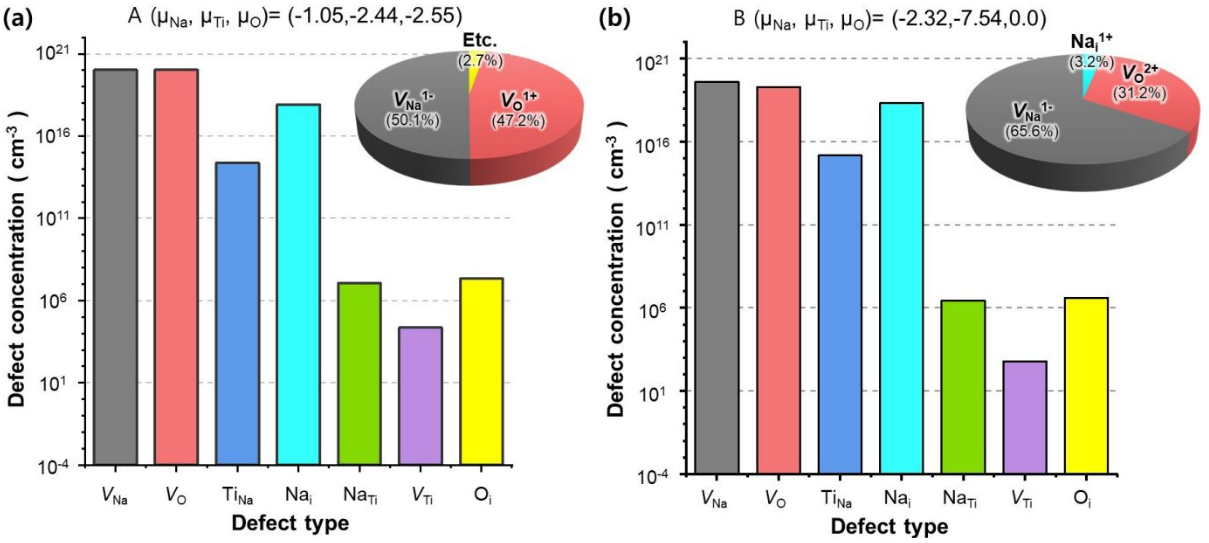

Figure S7. Equilibrium defect concentrations of $Na_2Ti_6O_{13}$ at the elemental chemical potential conditions of (a) A and (b) B in Figure 1b. Bar graphs are plotted in logarithm scale, whereas pie charts in the insets are in linear scale. All defect concentrations are calculated at 1070 K.

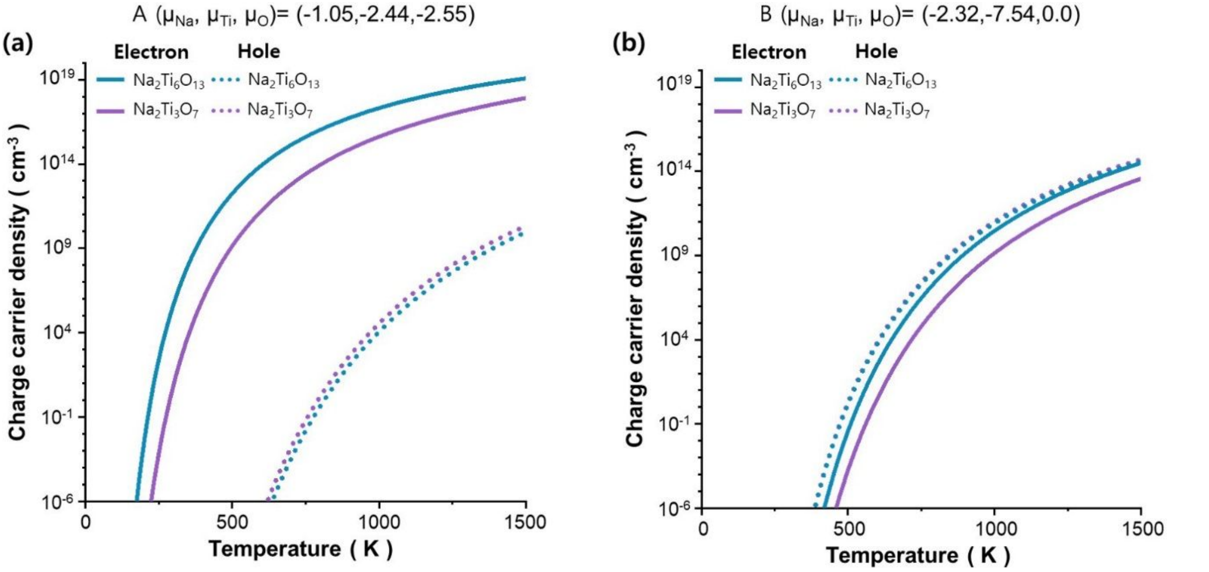

Figure S8. Comparison between the charge carrier densities of $Na_2Ti_3O_7$ and $Na_2Ti_6O_{13}$ at the elemental chemical potential conditions of (a) A and (b) B in Figure 1b. Regardless of temperature, $Na_2Ti_6O_{13}$ have higher concentration of electrons under oxygen-poor condition, suggesting higher electrical conductivity. Under oxygen-rich condition, both materials have lower charge carrier concentration compared to oxygen-poor condition, where $Na_2Ti_3O_7$ has slightly higher hole concentration than $Na_2Ti_6O_{13}$.

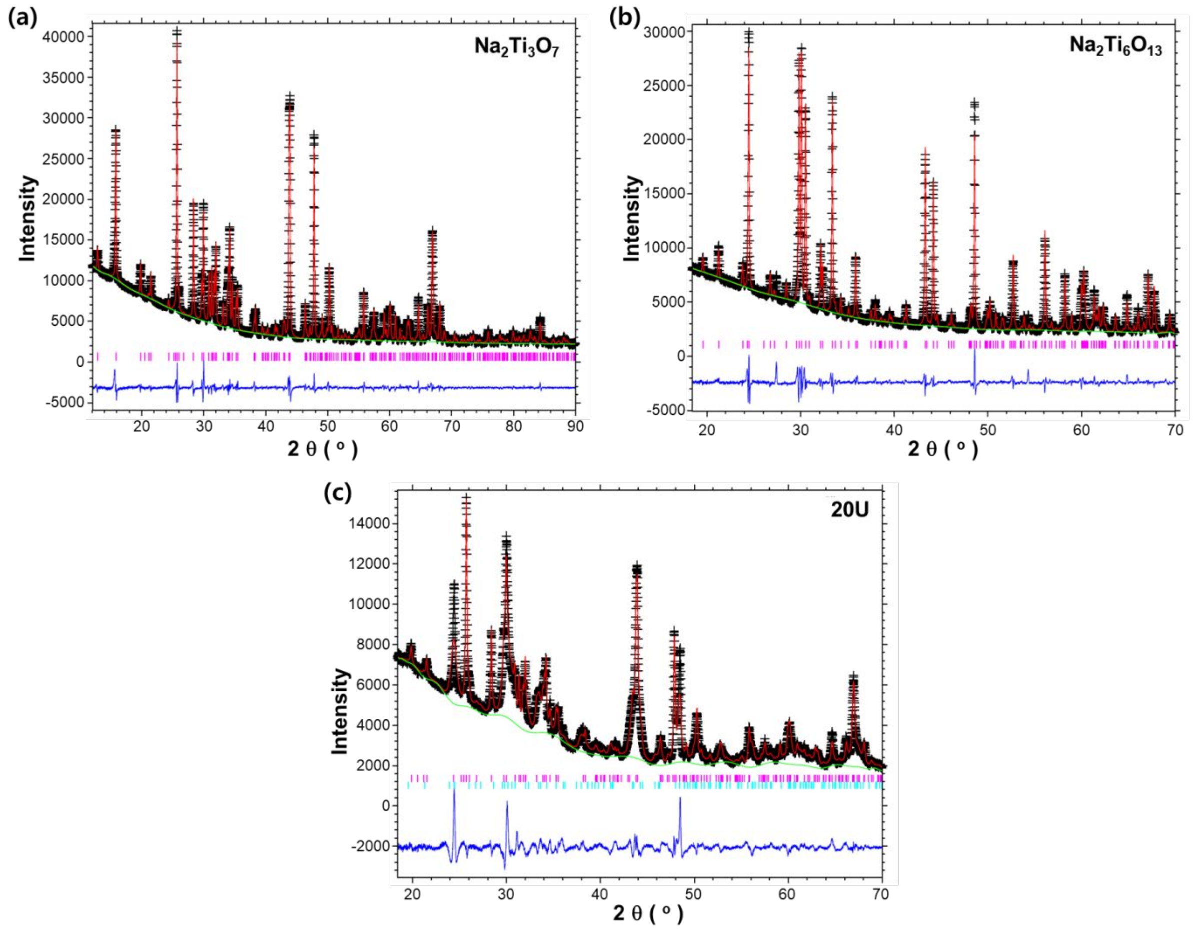

Figure S9. Rietveld refinement plots against lab XRD data of (a) $Na_2Ti_3O_7$, (b) $Na_2Ti_6O_{13}$, and the 20 wt% urea-treated titanate anode (20U) fitted using (a) $P12_1/m1$ $Na_2Ti_3O_7$ (ICSD 15463), (b) C2/m $Na_2Ti_6O_{13}$ (ICSD23877), and (c) a $Na_2Ti_3O_7$/$Na_2Ti_3O_7$ mixture with 49:51 wt% ratio, respectively. The black lines correspond to the observed data; the red lines indicate the calculated profiles, and the blue lines are the difference between the observed and calculated profiles. Purple tick marks in (a) and (b) indicate Bragg reflections of $Na_2Ti_3O_7$ and $Na_2Ti_6O_{13}$; purple and blue tick marks in (c) indicate Bragg reflections of $Na_2Ti_3O_7$ and $Na_2Ti_6O_{13}$.

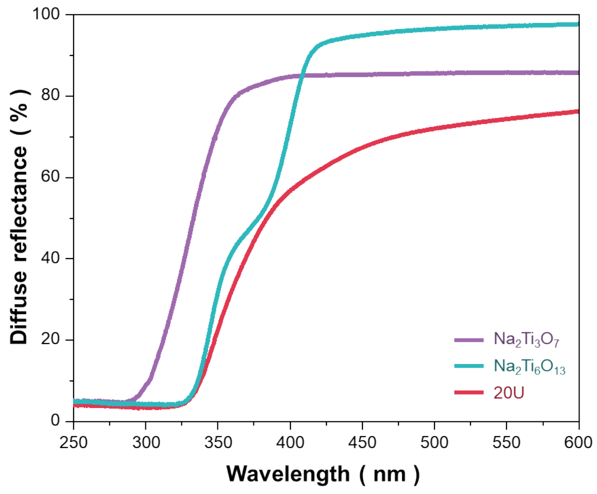

Figure S10. Ultraviolet-visible diffusive reflectance spectra measured in the range of 250 - 600 nm for $Na_2Ti_3O_7$, $Na_2Ti_6O_{13}$, and the 20 wt% urea-treated titanate anode (20U).

List of Tables

Table S1. List of lattice parameters (a, b, c) and angles (α, β, γ) of $Na_2Ti_3O_7$ and $Na_2Ti_6O_{13}$ optimized using PBEsol and HSE06 functionals. The lattice parameters a, b, and c are given in Å, whereas angles α, β, and γ are given in degrees. All structures are transformed into conventional ones for better comparison with experiments.

Phase	Methods	Lattice parameters (Å)			Angles (°)			Volume
		a	b	c	α	β	γ	(Å^3)
$Na_2Ti_3O_7$	PBEsol	8.4912	3.8108	9.1114	90	101.655	90	288.756
	HSE06	8.5239	3.7988	9.1294	90	101.559	90	289.623
	Exp.[1]	8.5624	3.7999	9.1245	90	101.592	90	290.829
	Exp.	8.5630	3.7996	9.1230	90	101.593	90	290.770
$Na_2Ti_6O_{13}$	PBEsol	15.1499	3.75304	9.20602	90	99.047	90	516.925
	HSE06	15.1228	3.74371	9.17649	90	98.849	90	513.348
	Exp.[29]	15.11	3.7467	9.1674	90	99.056	90	512.521
	Exp.	15.1042	3.7436	9.1701	90	99.043	90	512.078

Table S2. Thermodynamic transition levels (ε) of native defects in $Na_2Ti_6O_{13}$ obtained with respect to VBM. Note that most defects show deep ε(0/-) or ε(0/+) transition levels similar to those predicted from $Na_2Ti_3O_7$, preventing $Na_2Ti_6O_{13}$ to show polaronic conduction at room temperature. One exception is Ti_{Na} with its ε(0/+) transition level being positioned above CBM, but it is less likely to contribute to polaronic conduction as Ti_{Na} has the highest formation energy among all other donor defects (see Figure S5).

		ε (vs. VBM, eV)					ε (vs. CBM, eV)			
	(-3|-4)	(-2|-4)	(-2|-3)	(-1|-2)	(0|-2)	(0|-1)	(1|0)	(2|0)	(2|1)	(3|1)
$V_{Na,1}$						0.98				
$V_{O,1}$							0.51		1.19	
$V_{O,2}$							0.59		1.42	
$V_{O,3}$							0.42		0.67	
$V_{O,4}$							0.93		1.51	
$V_{O,5}$								0.8		
$V_{O,6}$							1.09		1.54	
$V_{O,7}$							0.53		0.9	
$V_{Ti,1}$	2.09		2.04	1.16		0.73				
$V_{Ti,2}$		1.54			1.23					
$V_{Ti,3}$	1.95		1.37	1.21		0.96				
$Na_{Ti,1}$			1.98	1.59		0.84				
$Na_{Ti,2}$			1.9	1.14		0.52				
$Na_{Ti,3}$			2.22		1.01					
$Na_{i,1}$							0.48			
$Na_{i,2}$							0.58			
$Na_{i,3}$							0.31			
$Na_{i,4}$							0.51			
$O_{i,1}$				2.00		1.27				
$O_{i,2}$				2.00		1.73				
$O_{i,3}$				2.82		1.22				
$O_{i,4}$				4.95		-0.64				
$Ti_{Na,1}$							-0.05			0.87

Table S3. Localization of polarons on ions neighboring native defects of $Na_2Ti_6O_{13}$. The site of localized hole/electron polarons are denoted in parenthesis. Oxygen dimer species formed upon DFT structural optimizations are also shown for reference.

	Polarons	Constituents
$Na_{Ti,1}$	0.61 (O4), 0.641 (O3), 0.623 (O7)	
$Na_{Ti,2}$	0.689 (O2), 0.689 (O2), 0.723 (O5)	
$Na_{Ti,3}$	0.71 (O1), 0.699 (O2), 0.733 (O3)	
$O_{i,1}$	1.389 (interstitial)	
$O_{i,2}$	1.502 (interstitial)	
$O_{i,3}$	1.389 (interstitial)	
$O_{i,4}$	0.588 (interstitial), 0.588 (O5), 0.249 (Ti2), 0.249 (Ti2)	Superoxide (O-Ti-O)
$V_{Na,1}$	0.682 (O2)	
$V_{Ti,1}$	0.623 (O4), 0.716 (O3), 0.663 (O4), 0.716 (O7)	
$V_{Ti,2}$	0.727 (O2), 0.727 (O2), 0.733 (O5), 0.75 (O7)	
$V_{Ti,3}$	0.717 (O2), 0.681 (O6), 0.765 (O1), 0.745 (O3)	
	Polarons	Constituents
$Na_{i,1}$	0.801 (Ti2)	
$Na_{i,2}$	0.834 (Ti2)	
$Na_{i,3}$	0.439 (Ti2), 0.446 (Ti2)	
$Na_{i,4}$	0.81 (Ti2)	
$Ti_{Na,1}$	1.723 (substitution), 0.829 (Ti2)	
$V_{O,1}$	0.848 (Ti2), 0.926 (Ti3)	
$V_{O,2}$	0.737 (Ti2), 0.737 (Ti2)	
$V_{O,3}$	0.998 (Ti1), 0.668 (Ti2)	
$V_{O,4}$	0.733 (Ti1), 0.733 (Ti1)	
$V_{O,5}$	0.843 (Ti2), 0.843 (Ti2)	
$V_{O,6}$	0.610 (Ti3), 0.549 (Ti3)	
$V_{O,7}$	0.937 (Ti1), 0.731 (Ti2)	

Table S4. Selected crystallographic data of pristine $Na_2Ti_3O_7$ and $Na_2Ti_6O_{13}$ resulted from the Rietveld refinement of the PXRD data at room temperature (Fig. S8).

**$Na_2Ti_3O_7$ (P12$_1$/m1)**							
Lattice parameters (Å)				Angles (°)			
a	b	c		α	β	γ	
8.563004	3.799599	9.123009		90.0	101.593	90.0	
Goodness of fit							
χ^2 = 6.834			R_{wp} = 3.65 %			R_p = 2.45 %	
Atom	Wyckoff position	Fractional coordinates x	y	z	Occupancy	100 x U_{iso} (Å^2)	
Na1	2e	0.498425	0.250000	0.156767	1.0	2.998	
Na2	2e	0.682913	0.250000	0.594279	1.0	3.398	
Ti1	2e	0.029027	0.250000	0.279834	1.0	1.363	
Ti2	2e	0.247466	0.67776	0.25	1.0	1.237	
Ti3	2e	0.144585	0.985269	0.25	1.0	1.021	
O1	2e	0.912991	0.039796	0.25	1.0	2.283	
O2	2e	0.800983	0.321094	0.25	1.0	0.747	
O3	2e	0.017265	0.751953	0.25	1.0	1.375	
O4	2e	0.324585	0.907482	0.25	1.0	3.33	
O5	2e	0.437854	0.647862	0.25	1.0	3.985	
O6	2e	0.217075	0.186017	0.25	1.0	1.948	
O7	2e	0.140986	0.461204	0.25	1.0	2.146	

**$Na_2Ti_6O_{13}$ (C2/m)**						
Lattice parameters (Å)				Angles (°)		
a	b	c		α	β	γ
15.10419	3.743628	9.170188		90	99.043	90
Goodness of fit						
χ^2 = 8.474			R_{wp} = 4.28 %			R_p = 2.8 %
Atom	Wyckoff position	Fractional coordinates x	y	z	Occupancy	100 x U_{iso} (Å^2)
Na1	4i	0.461872	0	0.268412	1.0	4.08
Ti1	4i	0.167911	0	0.436343	1.0	0.542
Ti2	4i	0.114521	0	0.097991	1.0	1.291
Ti3	4i	0.227499	0	0.770445	1.0	1.161
O1	4i	0.166828	0	0.916168	1.0	1.214
O2	4i	0.357202	0	0.883189	1.0	0.897
O3	4i	0.130225	0	0.613148	1.0	2.909
O4	4i	0.301736	0	0.569209	1.0	1.26
O5	2a	0	0	0	1.0	2.809
O6	4i	0.238568	0	0.243481	1.0	1.423
O7	4i	0.0702	0	0.291781	1.0	0.617

Table S5. Input parameters to AMSET used to predict electrical conductivity of $Na_2Ti_3O_7$ and $Na_2Ti_6O_{13}$.

$Na_2Ti_3O_7$	Parameters
Elastic constant (GPa)	$\begin{bmatrix} 110.37 & 68.51 & 62.34 & -0.06 & -0.07 & 6.74 \\ 68.51 & 226.16 & 70.57 & -0.11 & -0.01 & 2.13 \\ 62.34 & 70.57 & 152.19 & -0.04 & -0.06 & -23.77 \\ -0.06 & -0.11 & -0.04 & 35.12 & -7.91 & -0.02 \\ -0.07 & -0.01 & -0.06 & -7.91 & 50.31 & -0.02 \\ 6.74 & 2.13 & -23.77 & -0.02 & -0.02 & 39.04 \end{bmatrix}$
Piezoelectric constant	0.0
Polar optical phonon frequency (THz)	8.31
High frequency dielectric constant (ε_∞)	$\begin{bmatrix} 4.01 & 0.00 & -0.10 \\ 0.00 & 3.80 & 0.00 \\ -0.10 & 0.00 & 4.10 \end{bmatrix}$
Static dielectric constant (ε_0)	$\begin{bmatrix} 10.34 & 0.00 & -4.58 \\ 0.00 & 24.96 & 0.00 \\ -4.58 & 0.00 & 54.82 \end{bmatrix}$
Deformation potential (eV)	VBM= $\begin{bmatrix} 1.03 & 0.00 & 0.95 \\ 0.00 & 2.34 & 0.07 \\ 0.95 & 0.07 & 0.74 \end{bmatrix}$, CBM= $\begin{bmatrix} 2.32 & 0.01 & 1.48 \\ 0.01 & 0.30 & 0.03 \\ 1.48 & 0.03 & 1.18 \end{bmatrix}$
$Na_2Ti_6O_{13}$	Parameters
Elastic constant (GPa)	$\begin{bmatrix} 128.14 & 90.92 & 75.80 & 10.79 & 3.15 & 6.53 \\ 90.92 & 229.94 & 92.30 & -37.72 & -1.10 & 2.48 \\ 75.80 & 92.30 & 251.71 & -4.31 & 3.24 & 12.61 \\ 10.80 & -37.72 & -4.31 & 11.31 & -2.47 & 1.94 \\ 3.15 & -1.10 & 3.24 & -2.47 & 49.83 & -4.99 \\ 6.53 & 2.48 & 12.61 & 1.94 & -4.99 & 32.50 \end{bmatrix}$
Piezoelectric constant	0.0
Polar optical phonon frequency (THz)	8.57
High frequency dielectric constant (ε_∞)	$\begin{bmatrix} 4.13 & -0.01 & 0.01 \\ -0.01 & 4.16 & 0.00 \\ 0.01 & 0.00 & 4.60 \end{bmatrix}$
Static dielectric constant (ε_0)	$\begin{bmatrix} 22.85 & -1.46 & -3.30 \\ -1.46 & 28.83 & -0.81 \\ -3.30 & -0.81 & 56.91 \end{bmatrix}$
Deformation potential (eV)	VBM= $\begin{bmatrix} 1.17 & 0.16 & 0.56 \\ 0.16 & 1.26 & 0.30 \\ 0.56 & 0.30 & 0.70 \end{bmatrix}$, CBM= $\begin{bmatrix} 1.35 & 0.66 & 0.20 \\ 0.66 & 0.97 & 0.33 \\ 0.20 & 0.33 & 1.87 \end{bmatrix}$

Additional notes

In addition to its capability to predict the defect concentrations at synthesis temperature, defect formation energies can also provide insights on the number of mobile Na ions in sodium titanate anodes.[30] In case of $Na_2Ti_3O_7$, the Na stoichiometries calculated under the "frozen-in" assumption are $x_{Na,n}$ = 1.92 and $x_{Na,p}$ = 1.93 for oxygen-poor and oxygen-rich conditions (Figure 4), respectively, the number of which are often considered to be the mobile charge carrier populations. However, the mobility of Na^+ ions can be affected by Coulombic attractions and configurational entropies of other defect species, which may alter the concentration of mobile Na^+ ions.

1. **Effect of Na_{Ti} antisites**

Among intrinsic defects calculated in this study, one possible case that can lower the Na mobility is the Na_{Ti} antisites that traps Na^+ ions to vacant Ti site. The energy required for freeing these Na from $Na_2Ti_3O_7$, i.e., the Frenkel-pair formation energy for $Na_{Ti} \rightarrow Na_i + V_{Ti}$, ranges from 3.02 to 3.99 eV depending on the Ti site. In comparison, the Frenkel-pair formation energy for $Na_{Na} \rightarrow Na_i + V_{Na}$ corresponds to 4.19 eV ≤ ΔE ≤ 5.49 eV. The smaller energy cost of Frenkel-pair formation from Na_{Ti} antisites suggests that Na positioned on Ti sites can readily escape and thus, the migration of Na will not be significantly obstructed by Ti vacancies. However, the large energy cost of interstitial-vacancy recombination suggests that the self-diffusion of Na in $Na_2Ti_3O_7$ is rather sluggish, which may be one reason for the poor rate performance of $Na_2Ti_3O_7$ anodes.[1, 31, 32] Subsequent analyses on $Na_2Ti_6O_{13}$ revealed that the Frenkel-pair formation energies for both $Na_{Ti} \rightarrow Na_i + V_{Ti}$ and $Na_{Na} \rightarrow Na_i + V_{Na}$ are lower than $Na_2Ti_3O_7$ by 0.1 – 0.2 eV, which indicates the faster Na diffusion kinetics of secondary $Na_2Ti_6O_{13}$ as reported in literature.[33, 34]

2. **Effect of clustering of Na_i and V_{Na}**

Another factor that influences the mobility of Na is the clustering of Na interstitial and vacancies owing to their Coulombic attraction to each other. If this Coulombic interaction surpass the tendency for defects to be disordered state (manifested by configurational entropy), the Na_i and V_{Na} will bind together to form defect pairs and lowers the mobility of Na. The binding energy of the two defects can be estimated by the difference in energy before and after dissociation, which can be done by either comparing the energies between the closest and farthest Na_i–V_{Na} pairs or calculating dissociation energy of Na_i–V_{Na} pairs $\rightarrow Na_i + V_{Na}$. The preliminary calculations using PBEsol functional showed that the shortest Na_i–V_{Na} distance without recombination during geometry optimization was 4.0 Å whereas the largest separation of Na_i–V_{Na} pair possible in a 72-atom-supercell was 7.3 Å. Subsequent structural optimizations with HSE06 functionals revealed the difference in energy between these two supercells,

dissociation energy of Na_i–V_{Na} pair, was 1.01 eV. On the other hand, the dissociation energy calculated from Na_i–V_{Na} pairs $\rightarrow$ Na_i + V_{Na} gives 1.78, 2.09 and 3.09 eV for the dissociated Na interstitial in site 1, 2 and 3, respectively (Figure 2). Overall, the calculated dissociation energies are lower than the formation energies of individual Na_i and V_{Na} (2.10 – 2.75 eV) when Na interstitials are in between Ti–O layers ($Na_{i,1}$ and $Na_{i,2}$ in Figure 2), suggesting that Na_i and V_{Na} located in between Ti–O layers of $Na_2Ti_3O_7$ act as independent defects with disordered configurations. However, when Na interstitial is trapped inside Ti–O layers ($Na_{i,3}$ in Figure 2), these interstitials are difficult to be removed and thus, prefers the formation of Na_i–V_{Na} pair and impedes the Na migrations.

Above analyses on the Na mobility suggests that the clustering of Na_i–V_{Na} pair in $Na_2Ti_3O_7$ may reduce the number of mobile Na ions. The reduction in the Na mobility can lead to the fewer generation of $V_{Na,2}$–$V_{O,5}$ Schottky pairs, which results in the less phase transition from $Na_2Ti_3O_7$ to $Na_2Ti_6O_{13}$. From this perspective, we believe that the amount of precipitated secondary $Na_2Ti_6O_{13}$ phase during synthesis will be lower than those (7-8 %) predicted from Na stoichiometries in Figure 4.

References

1. Costa, S. I.; Choi, Y. S.; Fielding, A. J.; Naylor, A. J.; Griffin, J. M.; Sofer, Z.; Scanlon, D. O.; Tapia-Ruiz, N., Surface Engineering Strategy Using Urea To Improve the Rate Performance of Na2Ti3O7 in Na-Ion Batteries. *Chemistry–A European Journal* **2021,** *27* (11), 3875-3886.

2. Rietveld, H. M., A profile refinement method for nuclear and magnetic structures. *Journal of applied Crystallography* **1969,** *2* (2), 65-71.

3. Toby, B. H., EXPGUI, a graphical user interface for GSAS. *Journal of applied crystallography* **2001,** *34* (2), 210-213.

4. Kresse, G.; Hafner, J., Ab initio molecular dynamics for open-shell transition metals. *Physical Review B* **1993,** *48* (17), 13115.

5. Kresse, G.; Furthmüller, J., Efficiency of ab-initio total energy calculations for metals and semiconductors using a plane-wave basis set. *Computational materials science* **1996,** *6* (1), 15-50.

6. Anisimov, V. I.; Zaanen, J.; Andersen, O. K., Band theory and Mott insulators: Hubbard U instead of Stoner I. *Physical Review B* **1991,** *44* (3), 943.

7. Krukau, A. V.; Vydrov, O. A.; Izmaylov, A. F.; Scuseria, G. E., Influence of the exchange screening parameter on the performance of screened hybrid functionals. *The Journal of chemical physics* **2006,** *125* (22), 224106.

8. Perdew, J. P.; Burke, K.; Ernzerhof, M., Generalized gradient approximation made simple. *Physical review letters* **1996,** *77* (18), 3865.

9. Ceder, G.; Persson, K., The materials project: A materials genome approach. 2010.

10. Linstrom, P. J.; Mallard, W. G., The NIST Chemistry WebBook: A chemical data resource on the internet. *Journal of Chemical & Engineering Data* **2001,** *46* (5), 1059-1063.

11. Togo, A.; Tanaka, I., First principles phonon calculations in materials science. *Scripta Materialia* **2015,** *108*, 1-5.

12. Skelton, J. M.; Tiana, D.; Parker, S. C.; Togo, A.; Tanaka, I.; Walsh, A., Influence of the exchange-correlation functional on the quasi-harmonic lattice dynamics of II-VI semiconductors. *The Journal of chemical physics* **2015,** *143* (6), 064710.

13. Leung, W. W.; Savory, C. N.; Palgrave, R. G.; Scanlon, D. O., An experimental and theoretical study into NaSbS 2 as an emerging solar absorber. *Journal of Materials Chemistry C* **2019,** *7* (7), 2059-2067.

14. Buckeridge, J.; Scanlon, D. O.; Walsh, A.; Catlow, C. R. A., Automated procedure to determine the thermodynamic stability of a material and the range of chemical potentials necessary for its formation relative to competing phases and compounds. *Computer Physics Communications* **2014,** *185* (1), 330-338.

15. Ong, S. P.; Richards, W. D.; Jain, A.; Hautier, G.; Kocher, M.; Cholia, S.; Gunter, D.; Chevrier, V. L.; Persson, K. A.; Ceder, G., Python Materials Genomics (pymatgen): A robust, open-source python library for materials analysis. *Computational Materials Science* **2013,** *68*, 314-319.
16. Morgan, B. J., bsym: A basic symmetry module. *Journal of Open Source Software* **2017,** *2* (16), 370.
17. Freysoldt, C.; Grabowski, B.; Hickel, T.; Neugebauer, J.; Kresse, G.; Janotti, A.; Van de Walle, C. G., First-principles calculations for point defects in solids. *Reviews of modern physics* **2014,** *86* (1), 253.
18. Lany, S.; Zunger, A., Assessment of correction methods for the band-gap problem and for finite-size effects in supercell defect calculations: Case studies for ZnO and GaAs. *Physical Review B* **2008,** *78* (23), 235104.
19. Freysoldt, C.; Neugebauer, J.; Van de Walle, C. G., Fully ab initio finite-size corrections for charged-defect supercell calculations. *Physical review letters* **2009,** *102* (1), 016402.
20. Kumagai, Y.; Oba, F., Electrostatics-based finite-size corrections for first-principles point defect calculations. *Physical Review B* **2014,** *89* (19), 195205.
21. Lee, Y.-L.; Duan, Y.; Sorescu, D. C.; Saidi, W. A.; Morgan, D.; Thomas, K.; Epting, W. K.; Hackett, G.; Abernathy, H., Defect Thermodynamics and Transport Properties of Proton Conducting Oxide BaZr1− xYxO3− δ (x≤ 0.1) Guided by Density Functional Theory Modeling. *JOM* **2022,** *74* (12), 4506-4526.
22. Sharma, L.; Katiyar, N. K.; Parui, A.; Das, R.; Kumar, R.; Tiwary, C. S.; Singh, A. K.; Halder, A.; Biswas, K., Low-cost high entropy alloy (HEA) for high-efficiency oxygen evolution reaction (OER). *Nano Research* **2022,** *15* (6), 4799-4806.
23. Ren, X.; Wu, T.; Sun, Y.; Li, Y.; Xian, G.; Liu, X.; Shen, C.; Gracia, J.; Gao, H.-J.; Yang, H., Spin-polarized oxygen evolution reaction under magnetic field. *Nature communications* **2021,** *12* (1), 1-12.
24. Buckeridge, J., Equilibrium point defect and charge carrier concentrations in a material determined through calculation of the self-consistent Fermi energy. *Computer Physics Communications* **2019,** *244*, 329-342.
25. Faghaninia, A.; Ager III, J. W.; Lo, C. S., Ab initio electronic transport model with explicit solution to the linearized Boltzmann transport equation. *Physical Review B* **2015,** *91* (23), 235123.
26. Petousis, I.; Chen, W.; Hautier, G.; Graf, T.; Schladt, T. D.; Persson, K. A.; Prinz, F. B., Benchmarking density functional perturbation theory to enable high-throughput screening of materials for dielectric constant and refractive index. *Physical Review B* **2016,** *93* (11), 115151.

27. Le Bahers, T.; Rerat, M.; Sautet, P., Semiconductors used in photovoltaic and photocatalytic devices: assessing fundamental properties from DFT. *The Journal of Physical Chemistry C* **2014,** *118* (12), 5997-6008.
28. Rode, D., Low-field electron transport. In *Semiconductors and semimetals*, Elsevier: 1975; Vol. 10, pp 1-89.
29. Wu, C.; Wu, Z.-G.; Zhang, X.; Rajagopalan, R.; Zhong, B.; Xiang, W.; Chen, M.; Li, H.; Chen, T.; Wang, E., Insight into the origin of capacity fluctuation of Na2Ti6O13 anode in sodium ion batteries. *ACS applied materials & interfaces* **2017,** *9* (50), 43596-43602.
30. Squires, A. G.; Scanlon, D. O.; Morgan, B. J., Native defects and their doping response in the lithium solid electrolyte Li7La3Zr2O12. *Chemistry of Materials* **2019,** *32* (5), 1876-1886.
31. Xia, J.; Zhao, H.; Pang, W. K.; Yin, Z.; Zhou, B.; He, G.; Guo, Z.; Du, Y., Lanthanide doping induced electrochemical enhancement of Na 2 Ti 3 O 7 anodes for sodium-ion batteries. *Chemical science* **2018,** *9* (14), 3421-3425.
32. Chen, J.; Zhou, X.; Mei, C.; Xu, J.; Wong, C.-P., Improving the sodiation performance of Na2Ti3O7 through Nb-doping. *Electrochimica Acta* **2017,** *224*, 446-451.
33. Wu, C.; Hua, W.; Zhang, Z.; Zhong, B.; Yang, Z.; Feng, G.; Xiang, W.; Wu, Z.; Guo, X., Design and synthesis of layered Na2Ti3O7 and tunnel Na2Ti6O13 hybrid structures with enhanced electrochemical behavior for sodium-ion batteries. *Advanced science* **2018,** *5* (9), 1800519.
34. Hwang, J.; Cahyadi, H. S.; Chang, W.; Kim, J., Uniform and ultrathin carbon-layer coated layered Na2Ti3O7 and tunnel Na2Ti6O13 hybrid with enhanced electrochemical performance for anodes in sodium ion batteries. *The Journal of Supercritical Fluids* **2019,** *148*, 116-129.
